# MFG-E8 (LACTADHERIN): a novel marker associated with cerebral amyloid angiopathy

**DOI:** 10.1186/s40478-021-01257-9

**Published:** 2021-09-16

**Authors:** Paula Marazuela, Montse Solé, Anna Bonaterra-Pastra, Jesús Pizarro, Jessica Camacho, Elena Martínez-Sáez, H. Bea Kuiperij, Marcel M. Verbeek, Anna M. de Kort, Floris H. B. M. Schreuder, Catharina J. M. Klijn, Laura Castillo-Ribelles, Olalla Pancorbo, David Rodríguez-Luna, Francesc Pujadas, Pilar Delgado, Mar Hernández-Guillamon

**Affiliations:** 1 Neurovascular Research Laboratory, Vall d’Hebron Research Institute, Hospital Universitari Vall d´Hebron, Universitat Autónoma de Barcelona, Pg. Vall d´Hebron, 119-129, 08035 Barcelona, Spain; 2 Pathology Department, Vall d’Hebron University Hospital, Universitat Autònoma de Barcelona, Barcelona, Spain; 3 Department of Neurology, Donders Institute for Brain, Cognition and Behaviour, Radboud Alzheimer Centre, Radboud University Medical Center, Nijmegen, The Netherlands; 4 Department of Laboratory Medicine, Radboud University Medical Center, Nijmegen, The Netherlands; 5 Clinical Biochemistry Department, Vall d’Hebron University Hospital, Universitat Autònoma de Barcelona, Barcelona, Spain; 6 Stroke Unit, Department of Neurology, Vall d’Hebron University Hospital, Barcelona, Spain; 7 Neurology Department, Dementia Unit, Vall d’Hebron University Hospital, Barcelona, Spain

**Keywords:** Cerebral amyloid angiopathy, Alzheimer's disease, Laser capture microdissection, MFG-E8, Biomarkers, Cerebrospinal fluid

## Abstract

Brain accumulation of amyloid-beta (Aβ) is a crucial feature in Alzheimer´s disease (AD) and cerebral amyloid angiopathy (CAA), although the pathophysiological relationship between these diseases remains unclear. Numerous proteins are associated with Aβ deposited in parenchymal plaques and/or cerebral vessels. We hypothesized that the study of these proteins would increase our understanding of the overlap and biological differences between these two pathologies and may yield new diagnostic tools and specific therapeutic targets. We used a laser capture microdissection approach combined with mass spectrometry in the APP23 transgenic mouse model of cerebral-β-amyloidosis to specifically identify vascular Aβ-associated proteins. We focused on one of the main proteins detected in the Aβ-affected cerebrovasculature: MFG-E8 (milk fat globule-EGF factor 8), also known as lactadherin. We first validated the presence of MFG-E8 in mouse and human brains. Immunofluorescence and immunoblotting studies revealed that MFG-E8 brain levels were higher in APP23 mice than in WT mice. Furthermore, MFG-E8 was strongly detected in Aβ-positive vessels in human *postmortem* CAA brains, whereas MFG-E8 was not present in parenchymal Aβ deposits. Levels of MFG-E8 were additionally analysed in serum and cerebrospinal fluid (CSF) from patients diagnosed with CAA, patients with AD and control subjects. Whereas no differences were found in MFG-E8 serum levels between groups, MFG-E8 concentration was significantly lower in the CSF of CAA patients compared to controls and AD patients. Finally, in human vascular smooth muscle cells MFG-E8 was protective against the toxic effects of the treatment with the Aβ40 peptide containing the Dutch mutation. In summary, our study shows that MFG-E8 is highly associated with CAA pathology and highlights MFG-E8 as a new CSF biomarker that could potentially be used to differentiate cerebrovascular Aβ pathology from parenchymal Aβ deposition.

**Supplementary Information:**

The online version contains supplementary material available at 10.1186/s40478-021-01257-9.

## Introduction

Cerebral β-amyloidosis is characterized by the accumulation of amyloid-beta (Aβ) protein in the brain parenchyma and cerebral blood vessels, which are major features of Alzheimer’s disease (AD) and Aβ-associated cerebral amyloid angiopathy (CAA), respectively [1, 2]. AD is the main cause of dementia [3], whereas CAA is the most common cause of lobar intracerebral haemorrhage (ICH) in elderly individuals [4, 5]. The principal neuropathological hallmarks of AD are the presence of neuritic plaques, initiated by the deposition of Aβ in the neuropil, and the intracellular generation of neurofibrillary tangles (consisting of hyperphosphorylated tau protein) [6]. Conversely, CAA predominantly affects leptomeningeal and cortical arteries and arterioles, although capillaries are also frequently involved [7]. Population-based autopsy studies have estimated a CAA prevalence of 20–40% in non-demented individuals and 50–60% in those with dementia, which demonstrates its strong association with AD [8]. In fact, the prevalence of CAA pathology is particularly high in patients with AD, accounting for 47–100% of those patients [8].

Even though there is a large degree of overlap between the two pathologies, they are clinically distinct; whereas AD promotes neuronal loss and dementia, CAA leads to vascular dysfunction, lobar haemorrhage [9], and cognitive decline independent of AD [10]. Moreover, although Aβ accumulates in both pathologies, the mechanisms explaining the different localization of the deposits in the brain have not been fully elucidated. Some factors seem to favor vascular or parenchymal Aβ deposition, such as the length of Aβ peptides; Aβ40 is predominantly found in CAA-affected vessels, whereas Aβ42 is the main component of neuritic plaques in AD [11, 12]. In addition, mutations in the *APP* gene (β-amyloid precursor protein) can also predispose the formation and deposition of Aβ towards a vascular or a parenchymal location [13]. For instance, the E693Q mutation in *APP* causes the autosomal dominant disorder Dutch-type hereditary CAA (also known as hereditary cerebral haemorrhage with amyloidosis, Dutch type, HCHWA-D), which is characterized by severe Aβ deposition in cerebral and cortical arterioles with much less deposition in the brain parenchyma [9, 14]. On the other hand, a number of Aβ-associated proteins have been identified both in neuritic plaques and CAA, such as complement proteins, serum amyloid-P component, glycosaminoglycans, and apolipoprotein E (ApoE) or apolipoprotein J (ApoJ) [13]. Recently, via proteomic analysis, the presence of ApoE or ApoJ was confirmed in leptomeningeal and cortical vessels from CAA patients [15–17]. In these studies, other proteins were identified being specifically associated with vascular deposits and absent from neuritic plaques, such as tissue inhibitor of metalloproteinases‐3 (TIMP3) [15], norrin (NDP), collagen-α-2(VI) (COL6A2) [16], or sushi repeat-containing protein-1 (SRPX1) [18]. Whereas such a proteomic strategy has been widely used in AD models [19, 20], the identification of biomarkers related to vascular Aβ accumulation and their potential implications in the pathology have been less explored. Finding novel amyloid-associated proteins exclusively present in brain vessels is still a challenge and may be a valid approach to find novel biomarkers for CAA and provide new insights into the mechanisms of CAA formation. For this purpose, we used a laser capture microdissection approach combined with proteomics to selectively isolate vascular or parenchymal Aβ deposits from a transgenic mouse model of cerebral β-amyloidosis. We focused our study on milk fat globule-EGF factor 8 (MFG-E8), as it was one of the proteins specifically detected with Aβ-affected cerebral vessels and absent from parenchymal Aβ plaques in the APP23 mouse model of AD/CAA. MFG-E8, also known as lactadherin, is a secreted glycoprotein originally identified as a component of milk fat globules with multifunctional domains [21]. MFG-E8 accumulates within the arterial wall during aging and is abundantly expressed by vascular smooth muscle cells and other variety of cell types, including immune cells, astrocytes or microglia [22]. It has been reported to be involved in several physiological and pathophysiological processes, including immunity [23], angiogenesis [24, 25], and the clearance of apoptotic cells [26, 27] and platelet-derived microvesicles from the circulation [28, 29]. Moreover, MFG-E8 plays a role in Aβ42 phagocytosis in vitro [30, 31], although its contributions to vascular dysfunction in AD and CAA still need to be established.

The first aim of this study was to validate the expression and localization of MFG-E8 protein in the APP23 transgenic model. We next explored the cerebral distribution of MFG-E8 in human *postmortem* cortical sections from CAA patients, where its association with vascular and parenchymal Aβ deposition was examined. Furthermore, circulating MFG-E8 levels were analysed in serum and cerebrospinal fluid (CSF) from CAA patients, AD patients and healthy controls. Finally, the contribution of MFG-E8 to Aβ-induced cytotoxicity in cultured human vascular smooth muscle cells was also investigated.

## Material and methods

### APP23 transgenic mice model

Mice were maintained on a 12-h/12-h light–dark cycle in a temperature-controlled room with food and water available ad libitum. All animal procedures were approved by the Ethics Committee for Animal Experimentation of Vall d’Hebron Research Institute (78/13 CEEA) and were performed following Spanish legislation and in accordance with the Directives of the European Union. All experiments were carried out on APP23 transgenic male mice (hemizygote B6, D2-TgN [Thy-APPSWE]-23-Tg mice, The Jackson Laboratory, Bar Harbor, ME, USA) [32]. These transgenic mice overexpress the human-type APP protein with the Swedish mutation (K670M/N671L) under the control of the murine brain and neuron-specific Thy-1 promoter (thymocyte antigen-1). Hemizygous APP23 mice were backcrossed with C57BL/6 mice (Janvier Labs, Le Genest-Saint-Isle, France), and the APP genotype was tested by Transnetyx (Cordova, TN, USA). Male wild-type (WT) and APP23 mice were aged in the Vall d’Hebron Research Institute animal facility to obtain the final study cohort at different age points (12, 18 and 24 months). The number of mice per group is specified in each figure.

### Brain tissue preparation and laser capture microdissection

Paraffin-embedded brain tissue from 24-month-old APP23 and WT mice (n = 3/ group) was cut into 10 µm-thick sections and mounted onto ultraviolet-irradiated 2 µm polyethylene naphthalate (PEN) membrane slides (MicroDissect GmbH, Herborn, Germany). Between 10 and 20 brain sections from each mouse were used to isolate an approximate total area of 10^6^ µm^2^. An equivalent area of PEN membrane slices without brain tissue was also microdissected as negative control. Before microdissection, sections were deparaffinized for 1 h at 65 °C, rehydrated with graded ethanol solutions, and then stained with Thioflavin-S (ThS) (Sigma-Aldrich, Saint Louis, MO, USA) or tomato lectin (TL) (*Lycopersicon esculentum lectin*; Burlingame, Vector Labs, CA, USA) solution. ThS staining was performed to detect fibrillary Aβ (cerebral CAA vessels and parenchymal Aβ plaques) in APP23 mice. Briefly, sections were incubated in ThS (1% in 75% ethanol) for 30 s. The excess of ThS was removed, and the sections were then immersed in ThS (0.1% in 75% ethanol) for a minute. After washing with 75% ethanol, samples were dehydrated and air-dried. In parallel, sections from 24-month-old WT mice were stained with TL to label all cerebral blood vessels and to be used as control material. These sections were rinsed twice in PBS-1% Tween 20 (PBST) for 5 min and then incubated with DyLight 594-labelled TL diluted 1:100 in PBST for 1 h at room temperature (RT). Sections were finally washed with PBST, dehydrated, and air-dried. Laser capture microdissection (LCM) was performed using the 20 × objective of an LMD600 microscope (Leica Microsystems, Wetzlar, Germany). ThS-positive vessels or parenchymal plaques from APP23 mice and TL-positive cerebral vessels from WT mice were independently collected into the caps of 0.5 ml microcentrifuge tubes containing 20 μl of 1 mM EDTA (Sigma-Aldrich), 10 mM Tris (Sigma-Aldrich), 0.002% Zwitterionic detergent (Sigma-Aldrich) and 1/4 Protease Inhibitor Cocktail Tablet (Roche Diagnostic, Mannheim, Germany). The collected samples were frozen at − 80 °C until analysed.

### Liquid chromatography-mass spectrometry analysis and protein identification

Collected tissues were heated at 98 °C for 90 min and then sonicated for 60 min in a water bath refrigerated with ice. The samples were then digested with trypsin and subsequently analyzed using a linear ion trap Velos-Orbitrap mass spectrometer (Thermo Fisher, Bremen, Germany) by the Proteomics Unit of the Vall d’Hebron Institute of Oncology (VHIO, Barcelona, Spain). The peptide mixtures were fractionated by on-line nanoflow liquid chromatography using an EASY-nLC system (Proxeon Biosystems, Thermo Fisher) with a two-linear-column system. The LTQ Orbitrap Velos mass spectrometer was operated in the range of m/z 300 to 1600 with a resolution of 30,000. The 20 most abundant ions were selected for fragmentation by collision-induced dissociation with an isolation window of 2 Da.

Proteins were identified using a MASCOT search (Matrix Science, London, UK) against the SwissProt database (version 2016_0108) taxonomy selected for *Mus musculus* (16,754 entries) or *Homo sapiens* (20,194 entries). MS/MS spectra were searched with a precursor mass tolerance of 10 ppm, a fragment tolerance of 0.5 Da, and trypsin specificity with a maximum of two missed cleavages. The label-free approach was used for evaluation based on the total number of spectra identified for a single protein, known as the spectral count. All proteins were sorted in descending order according to the spectral count. The proteins identified in each group were considered present when they were detected in all three samples of each group with a spectral count ≥ 1. Shared proteins between experimental groups are also reported. All proteins identified for each experimental group are reported in Suppl. Data 1–7. The coverage of the murine MFG-E8 sequence and the peptides detected by mass spectrometry are shown in Additional file 1.

### Mouse brain homogenates

The soluble protein fraction was obtained from each hemisphere of 12-, 18- and 24-month-old APP23 and WT mouse brain. Brain tissue was homogenized with a Dounce homogenizer in 4 ml of Tris-buffered saline (TBS; pH 7.4) containing a cocktail of protease inhibitors (Roche). Each homogenate was then centrifuged at 8000 × g for 30 min at 4 °C. The supernatant was selected as the soluble protein fraction. The collected fractions were frozen at − 80 °C until use. The protein concentration of each fraction was quantified with a BCA Protein Assay kit (Thermo Fisher) and was used for the validation of MFG-E8 in brains from WT and APP23 mice by western blot analysis.

### Immunofluorescence in mouse brain sections

Immunofluorescence validation of MFG-E8 expression was performed in paraffin-embedded brain sections from 24-month-old APP23 and WT mice (n = 3/group). All samples were deparaffinized, hydrated, and treated with 70% formic acid for antigen retrieval for 30 min. The samples were blocked in 3% bovine serum albumin (BSA; Sigma-Aldrich) in PBST for 1 h at RT and then incubated overnight (ON) with goat anti-MFG-E8 antibody (1:50, R&D Systems, Minneapolis, MN, USA). After rinsing, samples were incubated with the secondary antibody anti-goat-Alexa Fluor-488 (Thermo Fisher) diluted 1:500 in blocking solution for 1 h at RT. Finally, samples were dehydrated, and 4′,6-diamidino-2-phenylindole (DAPI) was used for contrast staining before mounting the slices. MFG-E8-positive vessels were manually counted in each brain region under an Olympus BX61 microscope using a 10 ×  objective*.* Double immunofluorescence was performed for MFG-E8 and Aβ using the same procedure as previously described. A mouse anti-Aβ monoclonal antibody (4G8, 1:1000, BioLegend, San Diego, CA, USA) was used as the primary antibody, and anti-mouse Alexa Fluor-568 (1:500, Thermo Fisher) was used as the secondary antibody. Double immunofluorescence images were captured with an Olympus FV1000 confocal microscope.

### Immunohistochemistry in human brain tissue

*Postmortem* brain tissue was obtained from the Neurological Tissue Bank of the Vall d’Hebron University Hospital (VHUH). The study was performed on 6 cases neuropathologically diagnosed with CAA and 6 controls without CAA which were selected based on the absence of vascular amyloid pathology. Patients included in this study, or their relatives, expressed their willingness to donate brain tissue for research purposes. The project was approved by the Clinical Investigation Ethical Committee of the Vall d’Hebron University Hospital, PR(IR)173/2019. All samples were obtained from 2 to 20 h after death, and specimens were fixed in 10% buffered formalin for 3–4 weeks. All patients included in the study had a complete autopsy that included a neuropathological examination performed in the Pathology Department of the VHUH. Briefly, CAA lesions were graded following the criteria described by Greenberg and Vonsattel [33], and the classification into CAA type I and CAA type II was made according to the criteria described by Thal et al. [34]. AD neuropathologic change score was evaluated and staged according to the NIA-AA 2012 criteria [35], combining Thal Aβ phases [34], Braak (neurofibrillary tangle scores) [36] and CERAD’s criteria (neuritic plaque scores) [37]. Cases were then classified according to the level of AD neuropathologic change score (low, intermediate or high) previously defined [35].

The presence of Aβ and MFG-E8 immunostaining was evaluated in consecutive sections of previously selected paraffin blocks. Paraffin-embedded cortical brain sections were deparaffinized, hydrated, and incubated with citrate buffer to improve antigen exposure (10 mM sodium citrate, 0.05% Tween 20, pH = 6) for 30 min at 95 °C. The sections were then blocked in 10% goat serum in PBST for 1 h at RT and incubated ON with the anti-Aβ monoclonal antibody 4G8 (1:5000, BioLegend) or an anti-MFG-E8 polyclonal antibody (1:1000, Thermo Fisher). The slices were then treated with 3% hydrogen peroxide for 15 min to block endogenous peroxidases. Afterward, the slices were incubated with biotinylated anti-mouse IgG or anti-rabbit IgG (1:1000, Vector Labs) for 1 h at RT and then with streptavidin–horseradish peroxidase (HRP; 1:1000, Vector Labs). Finally, diaminobenzidine (DAB; Dako, Denmark) was applied to the samples, and the sections were immersed in Harris haematoxylin solution (Sigma-Aldrich) for contrast staining. The samples were dehydrated, and DPX (Sigma-Aldrich) was used as a mounting medium. A Pannoramic 250 scanner (3DHistech, Budapest, Hungary) was used with a 20 × objective to digitize the histological slides, and images were captured using Case Viewer Software (3DHistech). The immunodetection of Aβ and MFG-E8 in parenchymal plaques and cortical vessels was evaluated qualitatively according to the total number of deposits in an equivalent area of 385–400 mm^2^ in all the slides and was classified as follows: a score of 0 was assigned when no signal was detected (absence of deposits), a score of 1 was assigned for mild detection (1–50 deposits), a score of 2 was assigned for moderate detection (51–100 deposits), and a score of 3 was assigned for intense detection (> 100 deposits). The evaluation of the slides and the determination of the score were conducted by two investigators (P.M and E.M-S) blinded to the group distribution.

### Serum MFG-E8 determination

MFG-E8 levels were quantitatively determined by enzyme-linked immunosorbent assay (ELISA) in serum samples from CAA-associated ICH patients (n = 31), AD patients (n = 25), and age- and sex-matched controls (n = 39). All of them were recruited from the VHUH (Barcelona, Spain). The study was approved by the Clinical Investigation Ethical Committee of the Vall d’Hebron University Hospital, Barcelona, Spain (PR(AG)326/2014) and was conducted in accordance with the Declaration of Helsinki. All patients provided signed informed consent before inclusion. Serum samples from CAA patients were collected in the non-acute phase (at least two months after the last ICH) to avoid the initial inflammatory process. The CAA-ICH patients were > 55 years old and had suffered lobar ICH with clinical suspicion of CAA diagnosed by magnetic resonance imaging (MRI) following the modified Boston criteria [38]. The recruited AD patients presented sporadic probable AD according to the NIA-AA criteria [39]. The AD patients did not present a history of stroke before recruitment. The control subjects were healthy family members or companions of the patients with no history of stroke or dementia. Peripheral blood was collected in EDTA tubes, and serum was immediately separated by centrifugation and stored at −80 °C. The final results were analysed following the manufacturer’s instructions (R&D Systems, #DFGE80) in a Synergy™ Mx Microplate reader (BioTek Instruments Inc., Winooski, VT, USA). Each serum sample (fivefold dilution) was assayed in duplicate.

### Cerebrospinal fluid MFG-E8 determination

MFG-E8 levels in cerebrospinal fluid (CSF) were measured in two independent clinical patient cohorts. AD patients (n = 26) and age- and sex-matched controls (n = 10) were recruited from the VHUH (Barcelona, Spain). CAA patients (n = 23) and corresponding controls (n = 27) were recruited from the Radboud University Medical Center (Nijmegen, The Netherlands). The use of CSF samples from patients and controls from both cohorts (VHUV and Radboud UMC) was approved by the local medical ethical committees and informed consent was obtained from all study subjects or their legal representatives. CAA patients were diagnosed on the basis of the modified Boston Criteria [38], and were classified as either probable CAA (n = 18), CAA presenting with mixed-location cerebral hemorrhages (lobar and deep hemorrhages/microbleeds, n = 4)[38], or as confirmed suffering from a hereditary form of CAA, Hereditary Cerebral Hemorrhage With Amyloidosis-Dutch type patients (n = 1). The recruited AD patients presented sporadic probable AD according to the NIA-AA criteria [39] and did not present a history of stroke before recruitment. Control subjects underwent a lumbar puncture as part of the diagnostic workup of neurologic symptoms or to exclude central nervous system involvement of a systemic disease. They neither had the suspected neurological disease nor a neurodegenerative disease, known cognitive impairment, sepsis, stroke, nor a malignancy in the central nervous system (CNS). Additional inclusion criteria were the availability of a sufficient amount of CSF and a normal composition of the CSF for a number of routine parameters. The levels of t-tau, p-tau and Aβ42 in the CSF of controls and patients were previously obtained. CSF Aβ40 concentrations were only available from Radboud UMC patients and controls. The CSF biomarker profile and demographic characteristics for both control cohorts are described in Additional file 2.

All CSF samples were obtained by lumbar puncture via standard procedures, collected in polypropylene tubes, centrifuged, and stored at -80 °C. Finally, the CSF MFG-E8 levels were determined with a commercial ELISA kit (R&D Systems, #DFGE80) in tenfold diluted samples that were tested and analyzed in a blind manner to the diagnostic group.

### Cell culture and treatments

Primary cultures of human brain vascular smooth muscle cells (HBVSMCs) were obtained from Innoprot (Derio, Bizkaia, Spain). The cells were cultured in Dulbecco’s modified Eagle’s medium (DMEM, Sigma-Aldrich) supplemented with 10% fetal bovine serum (FBS; Gibco, MD, USA), 100 U/ml penicillin, and 100 μg/ml streptomycin and maintained at 37 °C in a humidified incubator containing 5% CO_2_. For treatments, cells were seeded on poly-L-lysine-coated 24- and 48-well cell culture plates at densities of 30,000 and 15,000 cells/ml, respectively. The HBVSMCs were grown in DMEM complete serum medium for 48 h and starved in FBS-free DMEM medium for the treatments. Aβ(1–40) and Aβ(1–40) containing the Dutch mutation (Aβ40-D; AnaSpec, San Jose, CA, USA) were dissolved in hexa-fluoro-2-isopropanol (Sigma-Aldrich) for 6 h and divided into 250 μg aliquots. The organic solvent was evaporated overnight, and aliquots were stored at −80 °C until use. To mimic the effect of Aβ in the cerebral vasculature, HBVSMCs were treated for 2 or 5 days with 25 μM Aβ(1–40) or 25 μM Aβ40-D previously resuspended in sterile water. Recombinant human MFG-E8 protein (rhMFG-E8; R&D Systems) was added at 2 µg/ml for 5 days.

### MFG-E8 knockdown by siRNA

HBVSMCs were seeded on poly-L-lysine-coated 48-well cell culture plates at a density of 15,000 cells/ml and were grown with DMEM complete serum medium for 48 h. A commercial Accell SMART pool of 4 short interfering RNAs (siRNAs, GE Dharmacon, Lafayette, CO, USA) was used to achieve MFG-E8 gene silencing. An Accell non-targeting siRNA pool (GE Dharmacon) was used as a negative control. The siRNAs were initially resuspended at 100 μM according to the manufacturer’s instructions. Cells were treated with 1 µM MFG-E8 siRNA or non-targeting siRNA. Forty-eight hours after transfection, fresh FBS-free medium was added, and the cells were then treated with 25 μM Aβ40-D for 2 days.

### Quantitative real-time reverse transcriptase-polymerase chain reaction (RT-qPCR)

Total RNA was isolated from HBVSMCs using a SpeedTools Kit (Biotools, Madrid, Spain), and cDNAs were synthesized through reverse transcription using a High-Capacity cDNA Reverse Transcription Kit (Applied Biosystems, Foster City, CA, USA) according to the manufacturer’s instructions. The mRNA levels were quantified using TaqMan probes for MFG-E8 (Hs00983890_m1, Thermo Fisher) and PPIA (peptidylprolyl isomerase A, Hs99999904_m1, Thermo Fisher). Quantitative real-time reverse transcription PCR (RT-qPCR) was performed in triplicate using an Applied Biosystems Prism 7900HT Fast System. Gene expression was normalized to PPIA levels, and relative quantification values were calculated using the Livak Eq. (2-ΔΔCt).

### Cell viability and total cell lysate preparation

The cytotoxicity in HBVSMCs was estimated by the mitochondrial-dependent reduction of 3-(4,5-dimethylthiazol-2-yl)-2,5-diphenyl tetrazolium bromide (MTT; Sigma-Aldrich) to purple formazan. Briefly, after treatments, cells were incubated with 0.5 mg/ml MTT solution for 90 min at 37 °C. The medium was replaced with dimethyl sulfoxide (Sigma-Aldrich) to dissolve formazan crystals, and the amount of formazan formed was spectrophotometrically quantified at 560 nm and 620 nm using a Synergy™ Mx Microplate reader (BioTek Instruments Inc.). Cell viability is expressed as a percentage of the control condition for each independent experiment.

Total cell lysates for western blot analysis were obtained by cell collection in freshly prepared ice-cold lysis buffer containing 50 mM Tris–HCl, 150 mM NaCl, 5 mM CaCl_2_, 0.05% BRIJ-35, 0.02% NaN_3_, 1% Triton X-100, 1% phenylmethanesulfonyl fluoride (Sigma-Aldrich), and 0.5% aprotinin (Sigma-Aldrich). The lysates were sonicated for 10 s and stored at -80 °C until use.

### Immunocytochemistry

HBVSMCs were grown on poly-L-lysine-coated glass coverslips at a density of 15,000 cells/ml for 2 days. The cells were fixed for 15 min with 4% paraformaldehyde and blocked with 3% BSA in PBST for 1 h at RT. The slices were then incubated overnight with the following primary antibodies diluted in blocking solution: rabbit anti-MFG-E8 (1:50, Thermo Fisher) and mouse anti-smooth muscle actin (1:100, SMA; Abcam, Cambridge, UK). After rinsing, the cells were incubated with secondary antibodies conjugated to Alexa Fluor 568 or Alexa Fluor 488 (Thermo Fisher) at a 1:500 dilution for 1 h at RT. Coverslips were finally mounted onto the glass slides using DAPI for nuceli staining. Images were acquired using a Zeiss LSM 980 confocal laser microscope.

### Western blot

The protein concentrations in cell lysates were determined using a Pierce BCA protein assay kit (Thermo Scientific). Equal amounts of protein from mouse membrane-bound brain fractions (50 μg) or cell lysates (10 μg) were analysed by 10% SDS-PAGE and transferred onto a nitrocellulose membrane using a Trans-Blot Turbo transfer system (Bio-Rad, Hercules, CA, USA). The membranes were blocked for 1 h with 10% non-fat milk in PBST, and the membranes were then incubated with specific primary antibodies at 4 °C ON. After rinsing, the membranes were incubated with HRP-labelled secondary antibodies for 1 h at RT. Finally, the protein bands were visualized by enhanced chemiluminescence using Pierce® ECL Western Blotting Luminol (Thermo Fisher). ImageJ software was used to quantify the intensity of specific bands. All protein measurements were divided by β-actin measurements for normalization. The primary antibodies used were goat anti-MFG-E8 (1:1000, R&D), rabbit anti-hMFG-E8 (1:1000, Thermo Fisher), and mouse anti-β-actin (1:10,000, Sigma-Aldrich). The following HRP-labelled secondary antibodies were used: anti-rabbit-HRP (1:2000, GE Healthcare Biosciences, Little Chalfont, UK), anti-mouse-HRP (1:2000, GE Healthcare Bioscience), and anti-goat-HRP (1:5000, GE Healthcare Bioscience).

### Statistical analyses

The SPSS 20.0 package (IBM Corporation, Armonk, NY, USA) and GraphPad Prism 6 (GraphPad Software, La Jolla, CA, USA) were used for statistical analysis. For experimental results, the Shapiro–Wilk test was used to analyse the normality of continuous variables. In animal experiments, significant differences between groups were determined by Student’s t-test. The effects of genotype (G) and age (A) were evaluated by two-way analysis of variance (ANOVA) with Bonferroni’s post hoc test. In human cell culture experiments, differences between groups were determined by one-way ANOVA with Tukey’s post hoc test for multiple comparisons. In studies based on human samples, descriptive statistics were used to define demographic data and clinical characteristics for the diagnostic groups (CAA patients and/or AD patients and controls). Contingency tables were constructed and chi-squared tests were conducted for categorical variables. The distributions of continuous variables were tested using the Shapiro–Wilk or Kolmogorov–Smirnov test, as appropriate. For normally distributed variables, one-way ANOVA with Tukey’s post hoc test was performed. For non-normally distributed variables, the Mann–Whitney U-test was used for one-to-one comparisons, and the Kruskal–Wallis test with Dunn’s post hoc test was used for multiple comparisons. Associations between two continuous variables were calculated with Spearman’s rho. The data are expressed as the mean ± SEM for normal distributions or as the median (interquartile range) for non-normal distributions. A *p*-value < 0.05 was considered to indicate statistical significance.

## Results

### Protein identification in the Aβ-affected cerebral vasculature

**Fig. 1:**
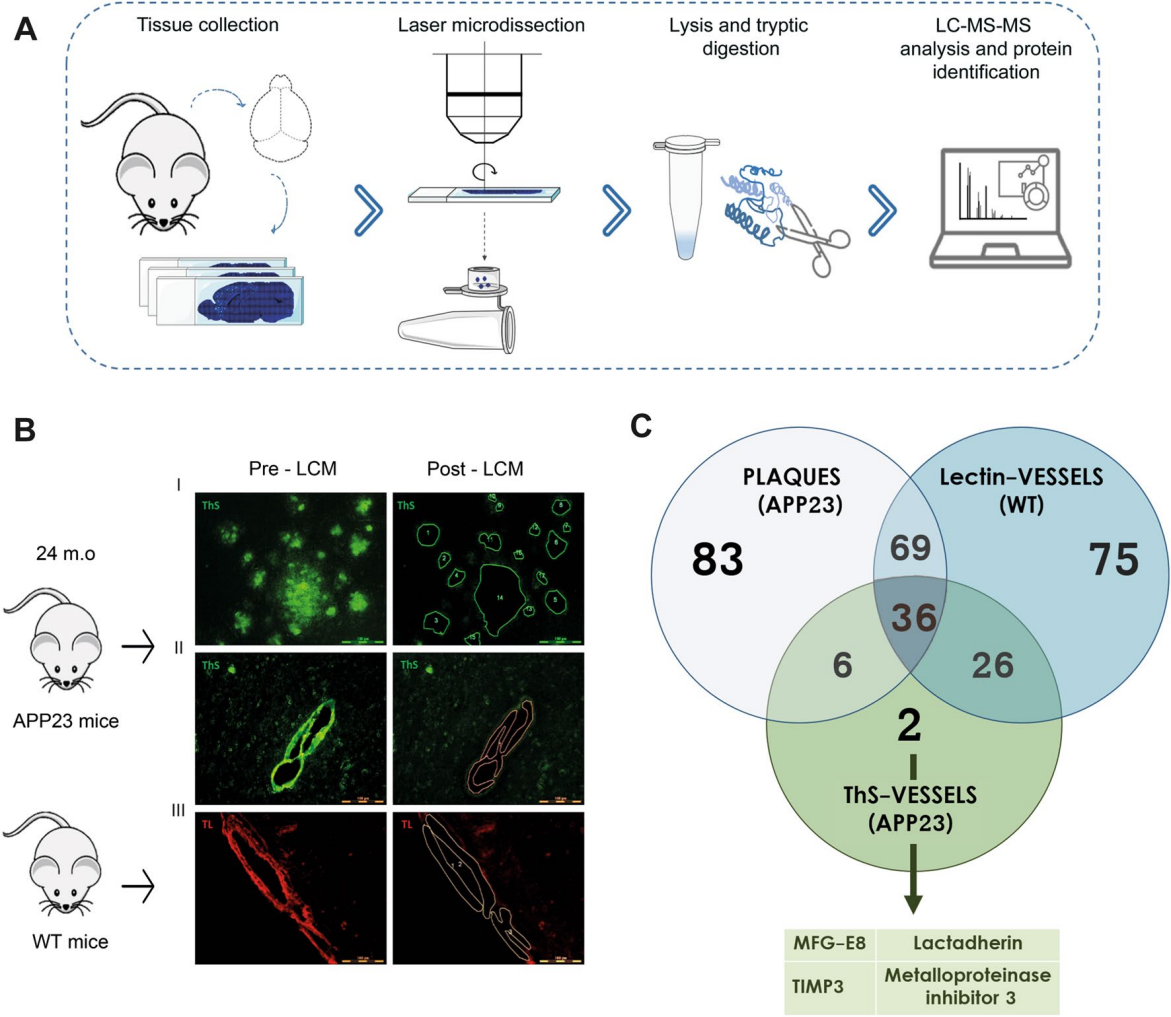
Study workflow for the identification of Aβ-associated proteins. **a** Schematic representation of the LCM proteomic approach for tissue collection, microdissection, data analysis, and protein identification. **b** Representative brain sections from 24-month-old APP23 and WT mice before and after LCM. I) Aβ plaques from APP23 brains stained with thioflavin S (ThS). II) Aβ-positive vessels from APP23 brains stained with ThS. III) Brain vessels from WT mice stained with tomato lectin (TL). The scale bar indicates 100 μm. **c** Venn diagram showing the total number of proteins identified in the three experimental groups

To identify proteins that specifically co-deposited with Aβ in the cerebrovasculature, we used LCM followed by mass spectrometry in consecutive paraffin-embedded brain sections from 24-month-old APP23 and WT transgenic mice (Fig. 1A). Amyloid plaques and Aβ-positive vessels were detected using ThS staining in APP23 brains and were independently microdissected, as shown in Fig. 1B. Cerebral blood vessels from WT mice were visualized using TL staining and were selected as control material for LCM isolation (Fig. 1B, III). The proteins identified in each group are described in Additional file 3. A total of 194 proteins associated with parenchymal Aβ deposits were identified in APP23 mice (Fig. 1C). Among them, 89 proteins were exclusively found in plaques, including well-known amyloid-associated proteins such as ApoE, ApoJ, and GFAP (Additional file 3). On the other hand, 70 proteins were found to be associated with Aβ-positive vessels. However, after exclusion of the proteins identified in parenchymal plaques from APP23 brains and in brain vessels from WT mice, the only proteins exclusively detected in the Aβ-affected cerebral vasculature were MFG-E8 and TIMP3 (Additional file 3). Because TIMP3 in cerebral vessels from CAA patients has previously been described [15], we focused our study on the other candidate, MFG-E8.

### MFG-E8 expression in brain tissue from APP23 transgenic mice

**Fig. 2:**
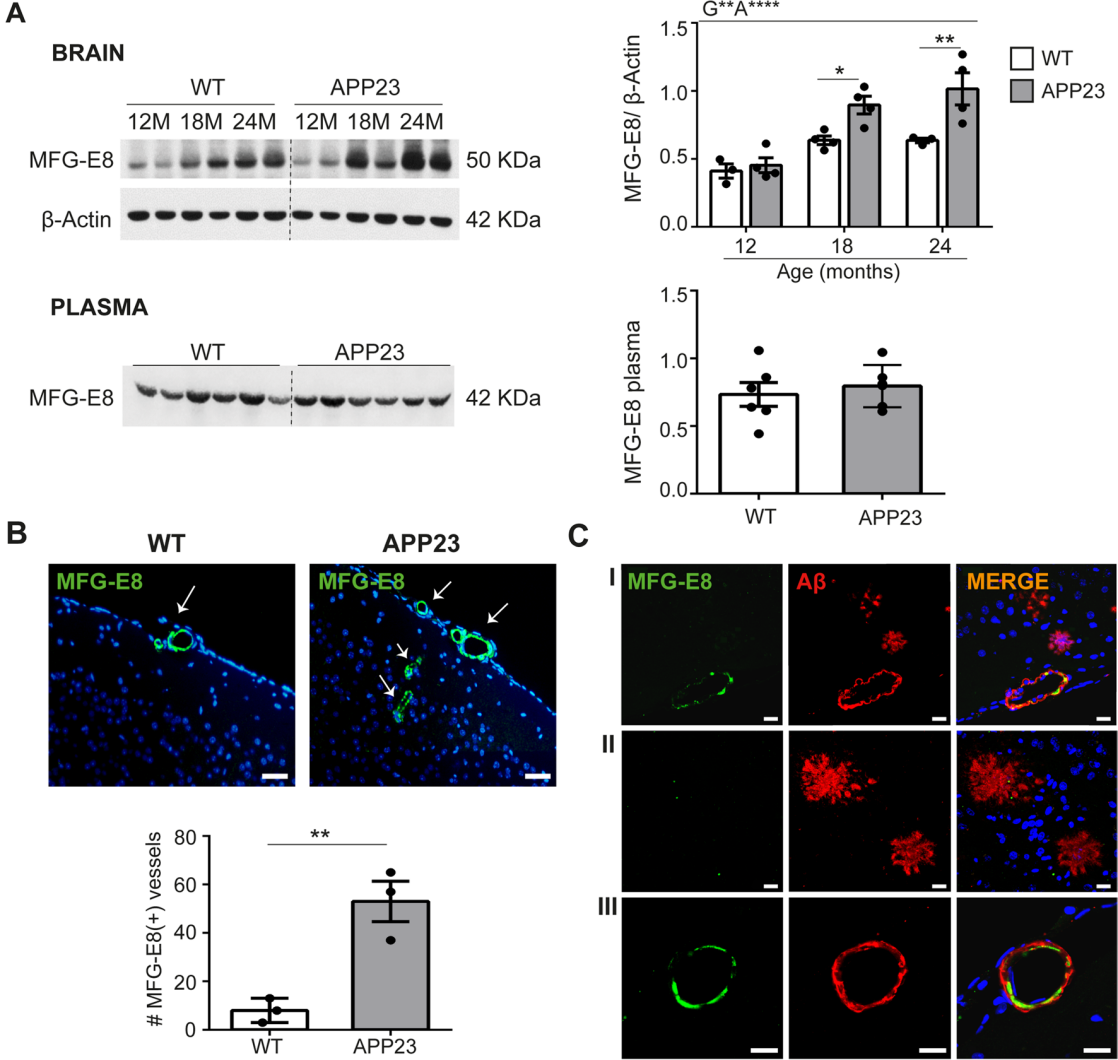
MFG-E8 expression analysis in APP23 mice. **a** Representative western blot images and quantification of MFG-E8 protein levels in brain homogenates from 12-, 18-, and 24-month-old WT and APP23 mice and in 24-month-old plasma samples, n = 3–6/group. **b** Representative immunofluorescence images and quantification of the MFG-E8-positive vessels in brains from 24-month-old WT and APP23 mice. The scale bar indicates 100 μm, n = 3/group. **c** Representative confocal images showing the localization of MFG-E8 (green) and Aβ (red) in parenchymal brain sections (I) from 24-month-old APP23 mice. II) Representative images showing no MFG-E8 detection in parenchymal Aβ deposits. III) Representative images showing MFG-E8 detection in an Aβ-positive vessel. The scale bar indicates 10 μm. *p < 0.05, **p < 0.01, ****p < 0.0001

To confirm the proteomic data, immunoblotting was performed on brain homogenates from 12- to 24-month-old mice. MFG-E8 brain levels were significantly higher in 18- and 24-month-old APP23 mice than in age-matched WT mice. Although MFG-E8 levels were strongly associated with genotype, an effect of age was also observed, only in APP23 mice (Fig. 2A). The plasma levels of MFG-E8 were also determined, although no significant differences between genotypes were found in 24-month-old mice (Fig. 2A). We next evaluated the distribution of MFG-E8 in the mouse brain by immunofluorescence staining. MFG-E8 was detected in brain vessels from both genotypes, but the number of MFG-E8-positive vessels was significantly higher in APP23 brains than in WT brains (Fig. 2B). Moreover, double immunofluorescence showed that MFG-E8 was detected principally in Aβ-positive vessels, and it was absent from Aβ plaques (Fig. 2C).

### MFG-E8 distribution in Aβ-positive cortical brain vessels from human CAA brains

**Fig. 3:**
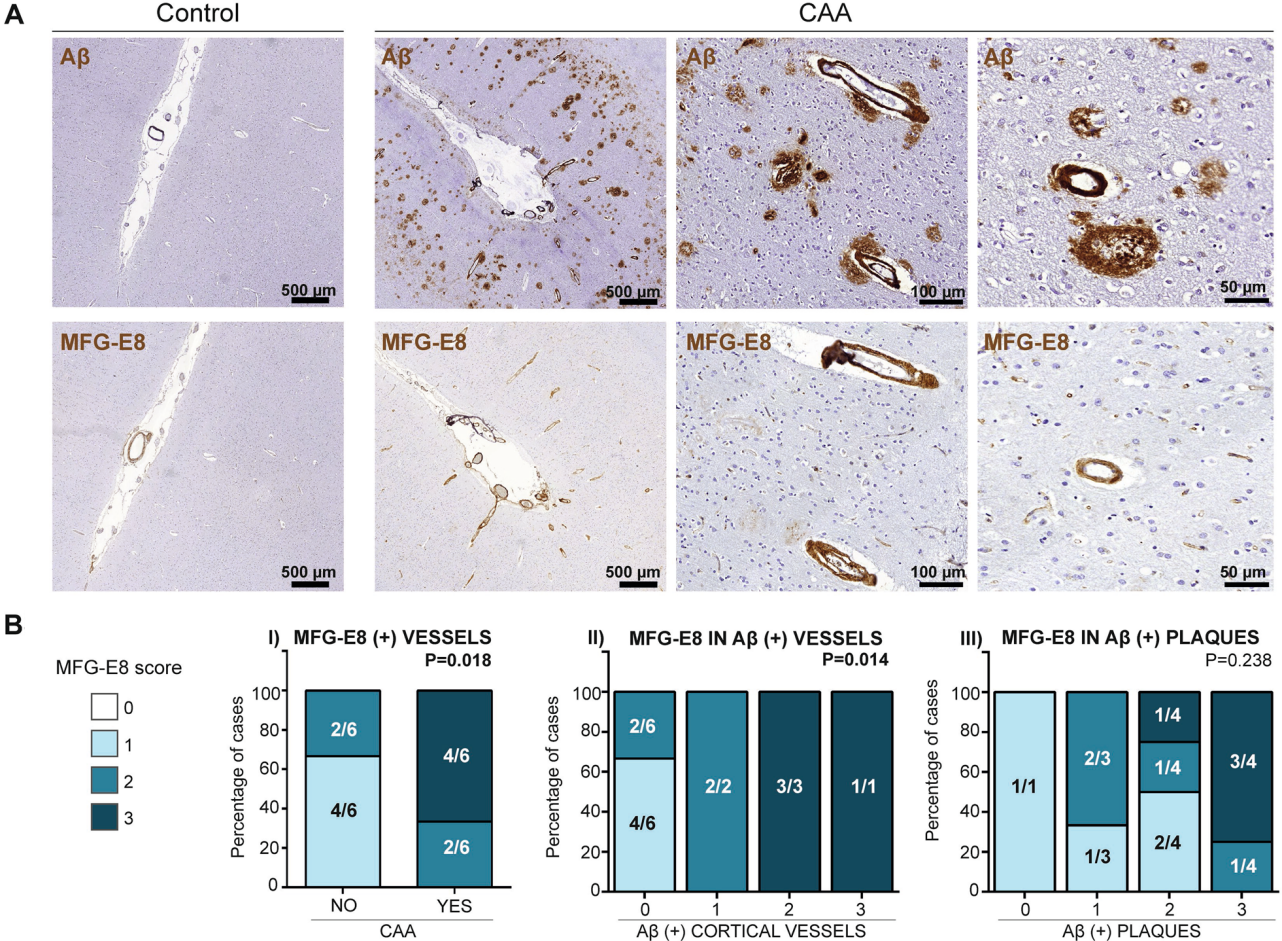
MFG-E8 immunodetection in cortical brain sections from CAA patients and controls. **a** Representative images of brain Aβ and MFG-E8 presence in consecutive cortical brain sections from CAA patients or control individuals. **b** Distribution of MFG-E8 staining according to (I) the presence or absence of CAA pathology and according to the (II) vascular and (III) parenchymal Aβ pathological burden. The graph shows the percentage of cases in each group. 0: No staining; 1: mild detection (1–50 positive deposits); 2: moderate detection (51–100); 3: intense detection (> 100 deposits) in the selected area for each section

Next, the expression of MFG-E8 in human *postmortem* cortical brain sections was analysed in patients diagnosed with pathological CAA and in a control cohort. The principal demographic characteristics, neuropathological features, and cause of death of all the cases included in the study are described in Additional file 4. Interestingly, strong MFG-E8 staining was detected only in cortical Aβ-positive vessels, as visualized in consecutive brain sections stained with anti-Aβ and anti-MFG-E8 in CAA cases (Fig. 3A). In fact, we confirmed the previous results found in APP23 mice in which MFG-E8 detection was restricted to vascular Aβ pathology, while Aβ parenchymal plaques did not show any immunoreactivity for MFG-E8. Control cases also showed some MFG-E8 immunoreactivity, although the staining was less pronounced in the brain parenchyma (Fig. 3A) and mostly restricted to leptomeningeal vessels.

The presence of MFG-E8 and Aβ in occipital brain sections was then evaluated and scored in the total cohort through IHC. The distribution of MFG-E8-positive vessels was significantly higher in CAA cases than in controls. Indeed, the highest MFG-E8 score was given to 66.6% of CAA cases (4/6), while the lowest scores were given to non-CAA cases (*p* = 0.018) (Fig. 3B, I). Regarding the distribution of MFG-E8 in Aβ-positive cortical vessels, significant differences were found between groups (*p* = 0.014). As expected, MFG-E8 immunodetection was strongest in those groups with the highest cerebrovascular Aβ scores; CAA cases with intense (1/1) or moderate (3/3) vascular Aβ deposition presented a MFG-E8 score of 3, while cases without vascular Aβ involvement presented a MFG-E8 score of 1 (4/6) or 2 (2/6) (Fig. 3B, II). On the other hand, CAA cases also presented an intense (4/6) or moderate (2/6) Aβ parenchymal deposition, however, the positivity of MFG-E8 in brain vessels was not significantly associated with the number of Aβ plaques (*p* = 0.238) (Fig. 3B, III). It is important to remark that MFG-E8 presence was neither associated with neuritic plaques nor diffuse parenchymal deposits. Age was significantly different among groups (CAA cases: 82.3 ± 6.4 yrs; control cases: 74.5 ± 5 yrs; *p* = 0.04), but the MFG-E8 immunostaining score was not associated with age or any other neuropathological features evaluated in the total cohort, including the Braak score or Vonsattel rating. MFG-E8 distribution was not associated with the AD neuropathological change score or the presence of ICH (Additional file 5).

### Circulating MFG-E8 levels

Circulating MFG-E8 levels were evaluated in serum samples from CAA-ICH patients (n = 31), AD patients (n = 25), and control participants (n = 39). However, no differences were found between groups (Additional file 6). Serum MFG-E8 levels were not associated with age, sex or other vascular risk factors (Additional file 7).

**Fig. 4:**
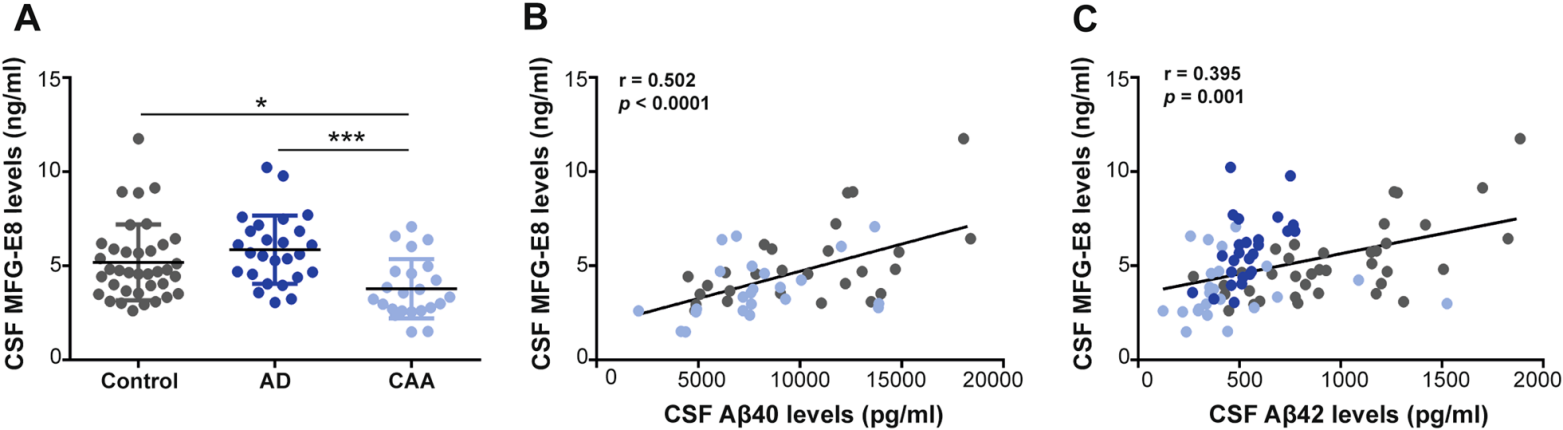
MFG-E8 levels in CSF from controls, AD patients, and CAA patients. **a** Analysis of CSF MFG-E8 levels in healthy controls (n = 37), AD patients (n = 26) and CAA patients (n = 23). **b** Correlation plot between CSF MFG-E8 levels and CSF Aβ40 levels among controls and CAA patients (n = 50). **c** Correlation plot between CSF MFG-E8 levels and CSF Aβ42 levels among controls, AD patients, and CAA patients (n = 86).*p < 0.05, ***p < 0.001. r, Spearman rho correlation coefficient

Circulating MFG-E8 levels were further evaluated in CSF samples from healthy controls, CAA patients, and AD patients obtained from two different clinical cohorts. Controls from Radboud UMC and from VHUH were pooled for posterior analysis (n = 37), as no significant differences were found regarding sex and age or levels of CSF biomarkers (p-Tau, t-Tau and Aβ42) between cohorts (Additional file 2). Demographics and levels of CSF markers from all the groups included in the study are shown in Table 1. CAA patients presented significantly lower MFG-E8 levels in CSF (3345.5 (2661.8–4648.3) pg/ml) than AD patients (5655.6 (4552.6–6849.2) pg/ml; p < 0.001) and control subjects (4569.4 (3533.9–5848.7) pg/ml; p = 0.01) (Fig. 4A). Differences remained significant after adjustment for age (CAA vs. controls: odds ratio (OR) 0.999 95% CI [0.999–1], p = 0.013; CAA vs. AD: OR 0.999 95% CI [0.999–1], p = 0.001). Next, the associations of MFG-E8 levels with other CSF biomarkers of the total cohort were evaluated. Interestingly, CSF MFG-E8 levels were positively correlated both with CSF Aβ40 (r = 0.502, p < 0.001) and Aβ42 levels (r = 0.395, p = 0.001) in the total cohort (Fig. 4B, C), suggesting that CSF MFG-E8 levels are dependent on Aβ levels. A weak correlation was also found between MFG-E8 levels and total tau levels in CSF (r = 0.236, p = 0.004). However, the concentration of the MFG-E8 protein in CSF was not associated with age (r = -0.121, p = 0.268) or CSF p-tau levels (r = 0.009, p = 0.932).

**Table 1:** Demographic characteristics and CSF parameters of healthy controls, CAA patients, and AD patients

	Control (n = 37)	CAA (n = 23)	AD (n = 26)	*p*-Value
*Demographics*				
Age, years, mean ± SD	63.8 ± 8.5	70.6 ± 7.8^**^	64.3 ± 7.3^$^	**0.004**
Sex (female), n (%)	11 (29.7%)	7 (30.4%)	14 (53.8%)	0.110
*CSF parameters, pg/ml*				
Aβ40, mean ± SD	10,187.9 ± 4009.1	7911.1 ± 3140.1	-	**0.032**
Aβ42, median (IQR)	895.1 (678–1225)	360 (317.5–462)^***^	514.6 (468.6–593.5)^***^	** < 0.001**
t-Tau, median (IQR)	231 (170–317)	403 (268–512.5)^**^	328.6 (183.4–395.3)	**0.002**
p-Tau, median (IQR)	28 (19–39)	45 (33.5–63.5)^**^	32.4 (18.1–39.6)^$^	**0.001**
MFG-E8, median (IQR)	4568.4 (3672.3–5898)	3345.5 (2661.8–4648.3)^*^	5655.6 (4552.6–6849.2)^$$$^	** < 0.001**

CAA, Cerebral amyloid angiopathy; AD, Alzheimer’s disease; CSF, cerebrospinal fluid; SD, standard deviation; IQR, interquartile range; -, not known. p-values below 0.05 are shown in bold: *p < 0.05 vs. the control group; **p < 0.01 vs. the control group; ***p < *0.001 vs. the control group; $p* < *0.05 vs. the CAA group; $$$ p* < *0.001 vs. the CAA group*

### Modulation of MFG-E8 expression by different Aβ-40 peptides in cultured HBVSMCs

**Fig. 5:**
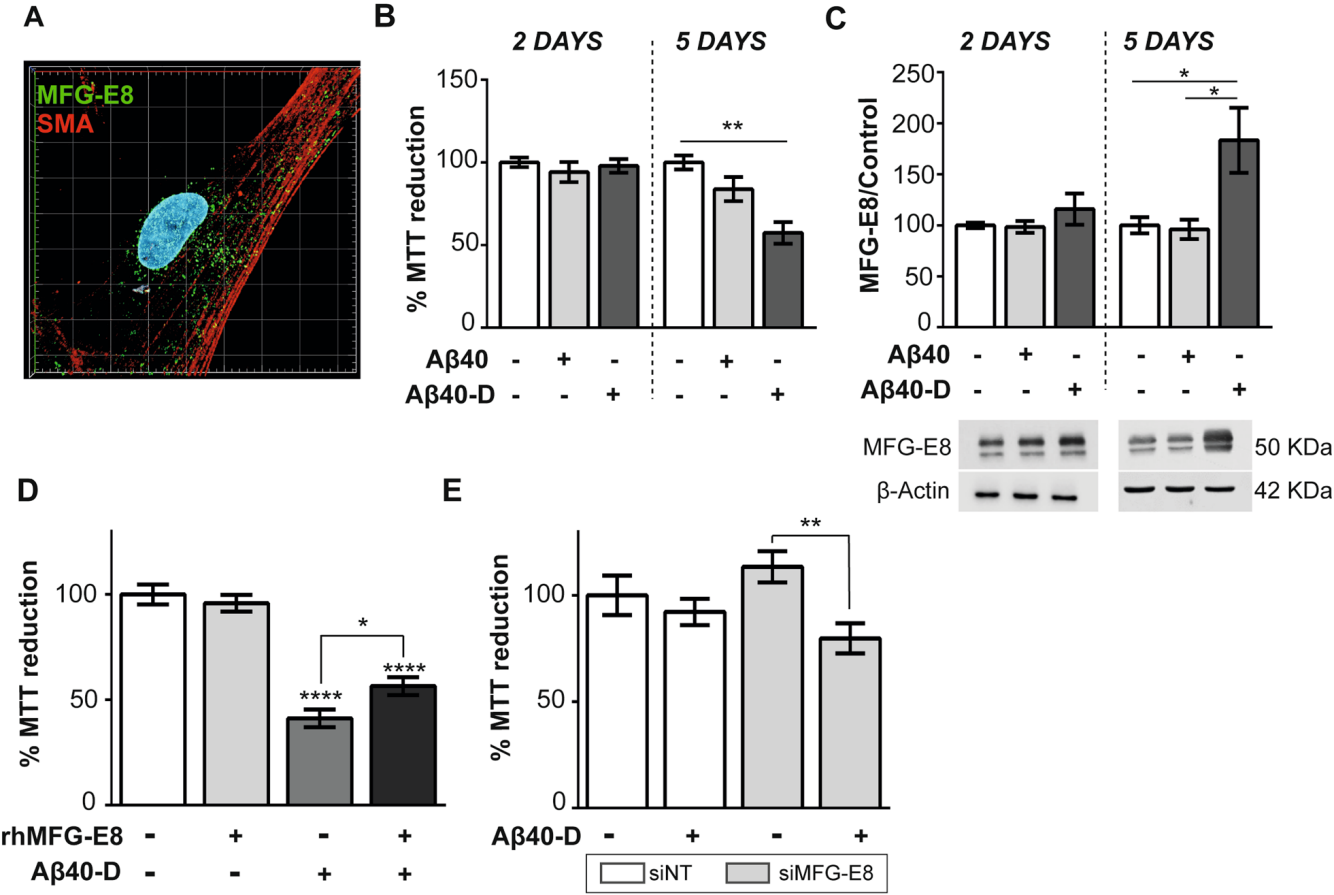
MFG-E8 expression in HBVSMCs after treatment with Aβ40 peptides. **a** Representative immunocytochemistry image showing the expression of MFG-E8 (green) and SMA (red) in HBVSMCs. **b** Cell viability after 2 and 5 days of treatment with 25 μM Aβ40 peptide and the toxic Aβ40-D peptide, n = 3–4/group. **c** Representative western blot images and quantification of MFG-E8 protein levels after 2 and 5 days of treatment with 25 μM Aβ40 peptide and the toxic Aβ40-D peptide, n = 3–4/group. **d** Cell viability of HBVSMCs co-treated with 2 µg/ml rhMFG-E8 and 25 μM Aβ40-D for 5 days (n = 5/group). **e** Cell viability of HBVSMCs treated with 25 μM Aβ40-D for 48 h after transfection with MFG-E8- or NT-siRNA (n = 5/group). A non-targeting siRNA (siNT) was used as a control. Significant differences among groups are indicated as * p < 0.05, ** p < 0.01, **** p < 0.0001

Vascular smooth muscle cells are widely known to express MFG-E8 [40]. Double immunostaining of MFG-E8 and SMA was performed to validate the in vitro model used, and confirmed the expression of MFG-E8 in HBVSMCs (Fig. 5A). The toxicity of two Aβ40 peptides, Aβ40-wt and Aβ40-D, on cell viability was determined. Whereas no cytotoxicity was induced by Aβ40 in brain vascular cells cultured under the conditions tested, a significant decrease in cell viability was observed after 5 days of treatment with the mutant Aβ40-D peptide (33.1% reduction *vs* control, *p* < 0.01) (Fig. 5B). Interestingly, MFG-E8 protein levels were significantly increased only in HBVSMCs after 5 days of treatment with the toxic Aβ40-D peptide (83.3% increase *vs* control, *p* = 0.04), (Fig. 5C). However, no differences in MFG-E8 expression were shown after treatment with the nontoxic Aβ40 peptide.

We next investigated whether exogenous administration of rhMFG-E8 was able to impact HBVSMC viability. Notably, no significant differences were found between non-treated cells and those treated with rhMFG-E8 (Fig. 5D), indicating that MFG-E8 was not cytotoxic to HBVSMCs under the conditions tested. However, co-administration of rhMFG-E8 protein and Aβ40-D peptide significantly reduced the Aβ40-D-induced cytotoxicity in HBVSMCs compared to cells treated with Aβ40-D alone (38.3% reduction in toxicity vs cells treated with Aβ40-D, p = 0.007) (Fig. 5D). Finally, to further confirm that MFG-E8 could play a protective role in CAA pathology by attenuating Aβ40-D cytotoxicity, MFG-E8 depletion was evaluated. HBVSMCs were treated with 1 μM MFG-E8-specific siRNA for 48 h and then subjected to 2 days of Aβ40-D treatment. The efficiency of MFG-E8 silencing was confirmed by RT-qPCR and western blot analyses 48 h post transfection (Additional file 8). Remarkably, MFG-E8 silencing exacerbated the slight Aβ40-D cytotoxicity observed at 2 days in HBVSMCs compared to that in non-transfected cells (14.1% increase in toxicity vs non-transfected cells treated with Aβ40-D, p < 0.001) (Fig. 5E).

## Discussion

Interest in understanding the pathological interactions between AD and CAA has grown considerably in recent years. The discovery of new markers related to cerebral Aβ deposition is crucial for a better comprehension of the underlying pathological mechanisms and could support the development of novel potential disease-modifying therapies. In the present study, we used an approach combining LCM and proteomics to identify new proteins specifically associated with vascular Aβ in a widely used transgenic mouse model of cerebral β-amyloidosis. Our proteomic data confirmed the presence of proteins already well known to be related to parenchymal and vascular Aβ deposits, such as ApoJ and ApoE [17, 41–43]. Remarkably, we identified two proteins selectively associated with cerebrovascular Aβ, TIMP3 and MFG-E8. We focused our study on the MFG-E8 candidate since TIMP3 has been previously described in vessels from CAA patients [15, 44]. Our proteomic results demonstrated that the LCM approach using an experimental transgenic model was a reliable tool that successfully validated candidates previously identified in other studies on humans.

MFG-E8 is a multifunctional glycoprotein expressed by a wide variety of cells, including epithelial cells, vascular smooth muscle cells, dendritic cells, microglia and astrocytes [22]. It has previously been associated with several physiological and pathological functions in the CNS, including phagocytosis of apoptotic cells [23, 26, 45], anti-inflammation [46, 47], and tissue regeneration[25], among others. In this regard, it has been shown that MFG-E8 binds to the Aβ42 peptide and increases microglial neuroprotective activity against oligomeric Aβ toxicity in vitro*,* suggesting that MFG-E8 can facilitate the clearance of Aβ by glial cells [30, 31]. The beneficial role of this protein in cerebral ischaemia has been extensively studied in mice, in which the treatment with MFG-E8 reduces the infarct volume and the expression of inflammatory cytokines [48, 49]. The anti-inflammatory and anti-apoptotic roles of MFG-E8 have also been described in MFG-E8-deficient mice with transient occlusion of the middle cerebral artery [50, 51] and in rat models of traumatic brain injury administered with rhMFG-E8 [52]. Additionally, a recent study also revealed that intra-cerebroventricular MFG-E8 administration after subarachnoid haemorrhage induction in mice decreases brain oedema by attenuating inflammation [53]. However, to the best of our knowledge, this is the first time that the MFG-E8 protein has been associated with vascular Aβ in CAA pathology.

In the CAA/AD experimental model, using immunofluorescence techniques, we detected an increased presence of MFG-E8 protein in Aβ-positive vessels from APP23 brains, while this protein was absent from parenchymal Aβ deposits, which supported the previous proteomic findings. Furthermore, we confirmed these results in human *postmortem* cortical brain tissues from CAA patients. Indeed, a strong association between MFG-E8 presence and Aβ-affected cortical vessels, but not the parenchymal Aβ burden, was observed. In fact, we found that MFG-E8 expression was not related to the neuropathologically assessed risk score for AD. A previous report using human *postmortem* brain samples showed that total mRNA MFG-E8 levels were decreased in AD brains compared to controls [30]. That study did not analyze gene expression in CAA brain samples and/or did not describe the prevalence of CAA pathology in the AD patient cohort, which makes it non-comparable to our study. However, it should be noted that low MFG-E8 protein expression in Aβ plaque-enriched areas was found, which would be in agreement with our results, in which no MFG-E8 immunoreactivity was detected in parenchymal Aβ deposits. On the other hand, the association of medin, a small fragment derived from MFG-E8 cleavage, with AD and vascular dementia has been recently described; previous studies have reported higher levels of medin in the cerebral arterioles from AD and vascular dementia patients than in those from controls [54]. Medin is one of the most common forms of ageing-related amyloid and accumulates in the vasculature, presenting a strong affinity to elastin fibres [55]. Increasing evidence suggests that medin may cause vascular dysfunction in ageing arteries and could contribute to age-associated vascular decline [56–58], although little is known about its physiological or pathologic effects and the mechanism by which it is cleaved from MFG-E8. In this line, a direct link between MFG-E8 and ageing has also been demonstrated [59–61]. However, in our study, MFG-E8 was not associated with age in the human cohorts analysed. Nevertheless, we detected MFG-E8 expression in specific vessels from healthy control subjects and in WT mice, and a significant association was found between MFG-E8 brain levels and ageing in APP23 mice when mice with a wide age range were included in the study. Taken together, the involvement of MFG-E8 expression in vascular ageing under physiological and pathological conditions merits further investigation.

Our results showing specific MFG-E8 accumulation in the Aβ-containing cerebral vessels raised questions regarding the cellular origin and the modulation of MFG-E8 protein expression. In this regard, because MFG-E8 was not detected in parenchymal deposits or expressed in microglia or other parenchymal cells, it is plausible to assume that the elevated presence of the protein could have resulted from its expression in cerebrovascular cells or from a peripheral source. To test this latter possibility, we analysed the circulating MFG-E8 levels in the serum of CAA patients. In fact, previous studies have described elevated circulating levels of MFG-E8 in patients with different inflammatory diseases [62, 63] and have observed an association between elevated MFG-E8 serum levels and the presence of high-intensity cerebral lesions on MRI [64]. Thus, we hypothesized that serum MFG-E8 may be modified in CAA patients, in whom the high prevalence of white matter lesions is well described [2, 65]. However, no differences were found in serum levels in CAA patients compared to AD cases or controls. In contrast, in CSF samples, lower MFG-E8 levels were found in the CAA group than in both the AD and control group. Interestingly, the Aβ CSF biomarker profiles for the clinical patient cohorts used in our study are consistent with previously reported data [66–68]. CAA patients presented lower Aβ42 and Aβ40 levels than controls. Remarkably, we found a positive correlation between MFG-E8 levels and Aβ40 and Aβ2 levels in the CSF, demonstrating, again, the strong association of this protein with Aβ levels. It has been hypothesized that CSF biomarker levels may reflect neuropathological changes in the brain [69, 70]. In this sense, the decreased Aβ levels in the CSF of CAA and AD patients may indicate possible impairment of the clearance of the peptides, potentially through the perivascular drainage pathway, which could explain the altered deposition of Aβ in the brain [71]. Similarly, our results demonstrate that MFG-E8 protein strongly accumulates in the cerebral vasculature of CAA patients, which may explain the reduced MFG-E8 concentrations in the CSF of these patients. In contrast, as MFG-E8 is absent from parenchymal Aβ deposits, no differences were observed in the CSF levels of MFG-E8 in AD patients. Further studies in independent cohorts prospectively recruited and provided with complete MRI data are needed to confirm whether measurement of CSF MFG-E8 levels could be used to differentiate cerebrovascular pathology from parenchymal Aβ deposition. Besides, future analysis may also elucidate whether CSF MFG-E8 levels could be combined with other biomarkers described in CSF for CAA, such as ApoD [72] or Aβ/p-Tau, to improve the accuracy of its diagnosis. Nevertheless, the biological overlap between CAA and AD pathologies involves an intrinsic limitation to differentiate the cohorts of the study. Although AD patients and controls selected in our study did not present a history of ICH, the lack of neuroimaging data in those cohorts could have masked the presence of CAA-related radiological markers. To overcome this limitation and define a clear CAA phenotype in comparison to other potential degrees of CAA pathology, we selected only patients with at least one lobar ICH or that had been clinically diagnosed according to the Boston clinical-radiographic criteria [38].

To determine the possible functional implication of MFG-E8 in CAA pathology, we simulated the pathological scenario by treating HBVSMCs with Aβ40, the most prominent peptide that accumulates in CAA, and Aβ40-D, the mutant form generated in HCHWA-D patients. Consistent with the findings of previous studies, only treatment with the Aβ40-D peptide induced a significant decrease in HBVSMC viability, confirming its highly toxic response [73, 74]. Several studies have shown that MFG-E8 is abundantly expressed in vascular smooth muscle cells [40, 55, 59]. In this regard, we found specific increases in MFG-E8 protein levels only after treatment with the toxic Aβ40-D peptide. Previous studies have shown that exogenous MFG-E8 significantly increases the expression of pro-inflammatory genes in vascular smooth muscle cells isolated from the thoracic aortas of aged mice [59]. However, under the conditions evaluated, cell viability was not altered after rhMFG-E8 treatment in human brain vascular smooth muscle cells. Indeed, we found that rhMFG-E8 supplementation significantly increased cell viability after Aβ40-D treatment and, complementarily, silencing of MFG-E8 increased Aβ40-D-induced toxicity. Altogether, these results suggest that MFG-E8 could play a protective role in CAA pathology, potentially reducing the cytotoxicity of Aβ. Whether MFG-E8 participates in Aβ phagocytosis or acts by attenuating the inflammation induced at the vascular level and reducing neuronal apoptosis requires further investigation.

In summary, this study covered different aspects of the relation of MFG-E8 with CAA pathology; we started with an experimental approach using a mouse transgenic model; then studied the levels of MFG-E8 in the brain, blood and CSF; and finally explored the effects of modulating MFG-E8 expression in cultured human smooth muscle cells. Overall, we have demonstrated that data obtained with an LCM proteomics approach using an experimental model of β-amyloidosis can be successfully translated to human studies. Our results indicate that MFG-E8 is closely associated with CAA; MFG-E8 levels are increased in cerebral Aβ-positive vessels but decreased in CSF from CAA patients. Finally, we have shown that MFG-E8 expression is increased in HBVSMCs by toxic Aβ variants, which may prevent the vascular damage induced by Aβ. The discovery of MFG-E8 as a specific novel marker for CAA opens up new opportunities to explore more accurate diagnostic tools and potential therapeutic approaches for CAA. Additional in vivo functional studies are required to elucidate the molecular pathways involved in the protective role of MFG-E8 in CAA pathology.

## Supplementary Information

**Additional file 1.** Domain structure and coverage of the murine MFG-E8 sequence and the peptides detected by mass spectrometry.

**Additional file 2.** Demographic characteristics and CSF parameters of controls from both cohorts.

**Additional file 3.** List of proteins found by mass spectrometry. Proteins are listed in decreasing order according to the number of spectral counts identified.

**Additional file 4.** Cause of death and patient demographic and neuropathological characteristics in cases with and without CAA.

**Additional file 5.** Univariate analysis of MFG-E8 distribution according to the demographic and neuropathological characteristics of the CAA brain cohort.

**Additional file 6.** Demographic and clinical characteristics of healthy controls, CAA-ICH patients, and AD patients.

**Additional file 7.** Univariate analysis of circulating MFG-E8 levels according to demographic and clinical characteristics of the total cohort.

**Additional file 8.** Western blot and qPCR results showing the gene silencing efficiency of the siRNA sequence targeting MFG-E8 in HBVSMCs.

## Abbreviations

AD: Alzheimer´s disease
ANOVA: Analysis of variance
ApoE: Apolipoprotein E
ApoJ: Apolipoprotein J
Aβ: Amyloid-beta
BSA: Bovine serum albumin
CAA: Cerebral amyloid angiopathy
CNS: Central nervous system
COL6A2: Collagen-α-2(VI)
CSF: Cerebrospinal fluid
DAB: Diaminobenzidine
DAPI: 4',6-Diamidino-2-phenylindol
DMEM: Dulbecco’s modified Eagle’s medium
ELISA: Enzyme-linked immunosorbent assay
FBS: Fetal bovine serum
HBVSMC: Human brain vascular smooth muscle cells
HCHWA-D: Hereditary cerebral haemorrhage with amyloidosis, Dutch type
HRP: Streptavidin–horseradish peroxidase
ICH: Intracerebral haemorrhage
LCM: Laser capture microdissection
MFG-E8: Milk fat globule-EGF factor 8
MRI: Magnetic resonance imaging
MTT: 3-(4,5-Dimethylthiazol-2-yl)-2,5-diphenyl tetrazolium bromid
NDP: Norrin
ON: Overnight
PBST: Phosphate buffered saline-1% Tween 20
PEN: Polyethylene naphthalate
PPIA: Peptidylprolyl isomerase A
rhMFG-E8: Recombinant human MFG-E8 protein
RT: Room temperature
RT-qPCR: Quantitative real-time reverse transcriptase-polymerase chain reaction
siRNA: Short interfering RNAs
SMA: Smooth muscle actin
SRPX1: Sushi repeat-containing protein-1
TBS: Tris-buffered saline
ThS: Thioflavin-S
TIMP3: Tissue inhibitor of metalloproteinases‐3
TL: Tomato lectin
VHUH: Vall d’Hebron University Hospital
WT: Wild-type
