# Supplementary material for: MFG-E8 (LACTADHERIN): a novel marker associated with cerebral amyloid angiopathy
Source: Acta Neuropathol Commun. 2021 Sep 16;9:154. doi: 10.1186/s40478-021-01257-9 (PMC8444498; doi:10.1186/s40478-021-01257-9)
Supplement: Supplementary file 1 — Additional file 1. Domain structure and coverage of the murine MFG-E8 sequence and the peptides detected by mass spectrometry. [file 40478_2021_1257_MOESM1_ESM.pdf]

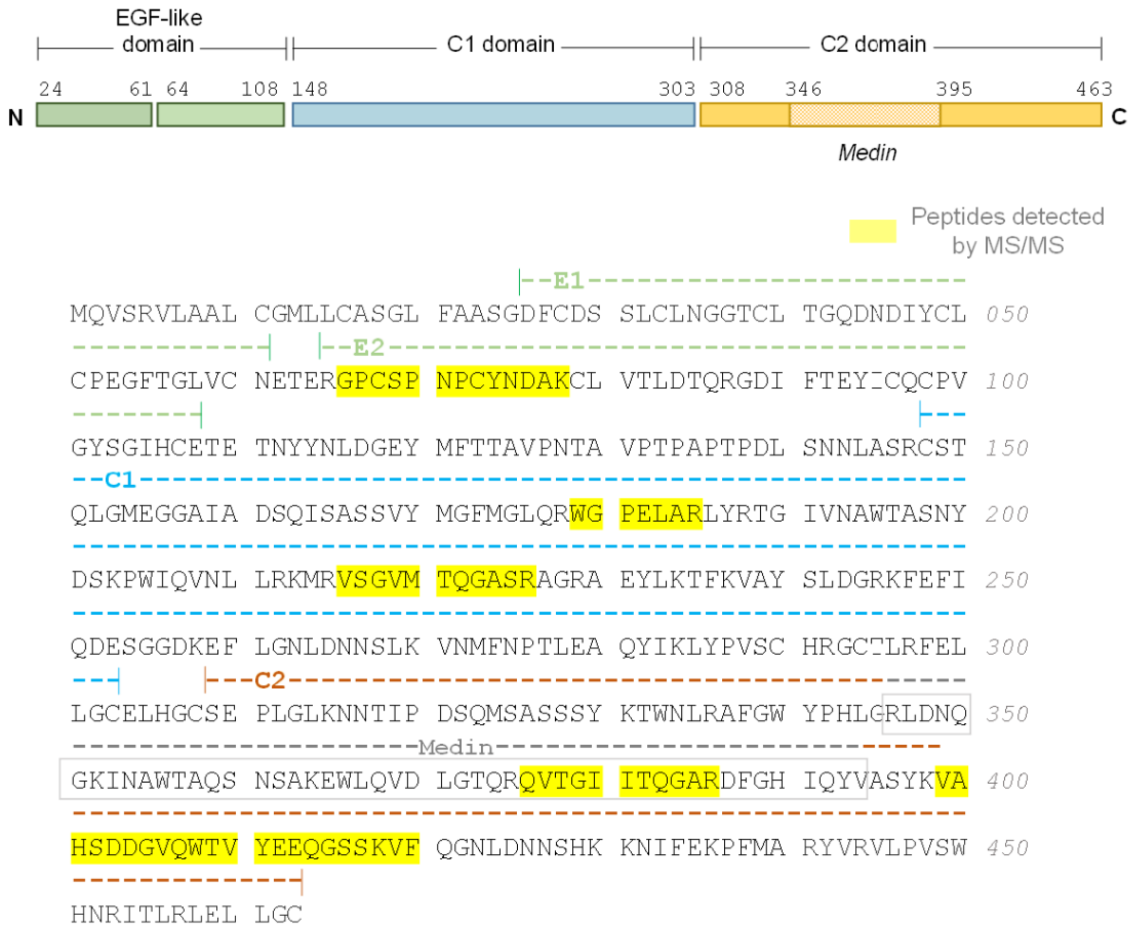

Domain structure and coverage of the murine MFG-E8 sequence and the peptides detected by mass spectrometry. Yellow blocks indicate sequences covered with peptides found in MS/MS runs.
