# Supplementary material for: MFG-E8 (LACTADHERIN): a novel marker associated with cerebral amyloid angiopathy
Source: Acta Neuropathol Commun. 2021 Sep 16;9:154. doi: 10.1186/s40478-021-01257-9 (PMC8444498; doi:10.1186/s40478-021-01257-9)
Supplement: Supplementary file 2 — Additional file 2. Demographic characteristics and CSF parameters of controls from both cohorts. [file 40478_2021_1257_MOESM2_ESM.pdf]

Demographic characteristics and CSF parameters of controls from both cohorts

	Radboud UMC Control (n=27)	VHUH Control (n=10)	*p*-Value	Total (n=37)
**Demographics**				
Age, years, mean ± SD	64.4 ± 8	62.1 ± 10.1	0.468	63.8 ± 8.5
Sex (female), n (%)	8 (29.6%)	3 (30.0%)	0.983	11 (29.7%)
**CSF parameters, pg/ml**				
Aβ42, median (IQR)	890 (586-1222)	905.3 (777-1225)	0.846	895.1 (678-1225)
t-Tau, median (IQR)	248 (200-327.5)	188.7 (137.7-218.2)	0.718	231 (170-317)
p-Tau, median (IQR)	37 (27-42)	13.4 (11.3-18.0)	0.120	28 (19-39)
MFG-E8, median (IQR)	4569.4 (3533.9-5848.7)	5257.2 (4440.1-6193.3)	0.378	4568.4 (3672.3-5898)

Abbreviations: CSF, cerebrospinal fluid; SD, standard deviation; IQR, interquartile range; UMC, University Medical Center; HVUH, Vall d'Hebron University Hospital
