# Supplementary material for: MFG-E8 (LACTADHERIN): a novel marker associated with cerebral amyloid angiopathy
Source: Acta Neuropathol Commun. 2021 Sep 16;9:154. doi: 10.1186/s40478-021-01257-9 (PMC8444498; doi:10.1186/s40478-021-01257-9)
Supplement: Supplementary file 3 — Additional file 3. List of proteins found by mass spectrometry. Proteins are listed in decreasing order according to the number of spectral counts identified. [file 40478_2021_1257_MOESM3_ESM.pdf]

Supplementary Data: List of proteins found by mass spectrometry. Proteins are listed in decreasing order according to the number of spectral counts identified. **(1)** Detected proteins in Aβ plaques. **(2)** Detected proteins in WT brain vessels. **(3)** Detected proteins in Aβ-positive vessels. **(4)** Common identified proteins between Aβ plaques and Aβ vessels. **(5)** Common identified proteins between WT brain vessels and Aβ vessels. **(6)** Common identified proteins between Aβ plaques and WT brain vessels. **(7)** Common identified proteins among the three experimental groups.

Supplementary Data 1. Detected proteins in Aβ plaques

#	Accession	Description
1	TBB2B	Tubulin beta-2B chain
2	TBB4A	Tubulin beta-4A chain
3	AT1A3	Sodium/potassium-transporting ATPase subunit alpha-3
4	TBB3	Tubulin beta-3 chain
5	APLP2	Amyloid-like protein 2
6	LRP1	Prolow-density lipoprotein receptor-related protein 1
7	STXB1	Syntaxin-binding protein 1
8	NSF	Vesicle-fusing ATPase
9	KCC2A	Calcium/calmodulin-dependent protein kinase type II subunit alpha
10	SNP25	Synaptosomal-associated protein 25
11	EAA2	Excitatory amino acid transporter 2
12	STX1B	Syntaxin-1B
13	NRX1A	Neurexin-1
14	DYN1	Dynamin-1
15	PGAM1	Phosphoglycerate mutase 1
16	CYTC	Cystatin-C
17	MAP6	Microtubule-associated protein 6
18	EAA1	Excitatory amino acid transporter 1
19	PP2BA	Serine/threonine-protein phosphatase 2B catalytic subunit alpha isoform
20	SPON1	Spondin-1
21	VPP1	V-type proton ATPase 116 kDa subunit a isoform 1
22	SYN1	Synapsin-1
23	VATB2	V-type proton ATPase subunit B, brain isoform
24	DYHC1	Cytoplasmic dynein 1 heavy chain 1
25	RAB3A	Ras-related protein Rab-3A
26	MYPR	Myelin proteolipid protein
27	OLFL3	Olfactomedin-like protein 3
28	SYT1	Synaptotagmin-1
29	VA0D1	V-type proton ATPase subunit d 1
30	THY1	Thy-1 membrane glycoprotein
31	GBB2	Guanine nucleotide-binding protein G(I)/G(S)/G(T) subunit beta-2
32	VAMP2	Vesicle-associated membrane protein 2
33	MYH10	Myosin-10
34	SEPT7	Septin-7
35	APOD	Apolipoprotein D
36	ARL8A	ADP-ribosylation factor-like protein 8A
37	C1QC	Complement C1q subcomponent subunit C
38	C1QT4	Complement C1q tumor necrosis factor-related protein 4
39	CNTN1	Contactin-1
40	G6PI	Glucose-6-phosphate isomerase
41	NDRG2	Protein NDRG2
42	PCSK1	ProSAAS
43	SNAB	Beta-soluble NSF attachment protein
44	STX1A	Syntaxin-1A
45	SYPH	Synaptophysin
46	VISL1	Visinin-like protein 1
47	PYGB	Glycogen phosphorylase, brain form
48	AMPH	Amphiphysin
49	CAPZB	F-actin-capping protein subunit beta
50	COR1A	Coronin-1A
51	GPC1	Glypican-1
52	GUAD	Guanine deaminase
53	HEXB	Beta-hexosaminidase subunit beta
54	RAB7A	Ras-related protein Rab-7a
55	TENR	Tenascin-R
56	ACBP	Acyl-CoA-binding protein
57	CAPG	Macrophage-capping protein
58	KCD12	BTB/POZ domain-containing protein KCTD12
59	RAC1	Ras-related C3 botulinum toxin substrate 1
60	EPDR1	Mammalian ependymin-related protein 1
61	GDN	Glia-derived nexin
62	STX7	Syntaxin-7
63	TPPP	Tubulin polymerization-promoting protein
64	TSN7	Tetraspanin-7
65	UBE2N	Ubiquitin-conjugating enzyme E2 N
66	PEA15	Astrocytic phosphoprotein PEA-15
67	AT1B2	Sodium/potassium-transporting ATPase subunit beta-2
68	GPC4	Glypican-4
69	GPC5	Glypican-5
70	HSP74	Heat shock 70 kDa protein 4
71	LSP1	Lymphocyte-specific protein 1
72	MK	Midkine
73	PDC6I	Programmed cell death 6-interacting protein
74	PRIO	Major prion protein
75	PROF2	Profilin-2
76	RAB14	Ras-related protein Rab-14
77	RAB2A	Ras-related protein Rab-2A
78	SDCB1	Syntenin-1
79	SH3G1	Endophilin-A2
80	SKP1	S-phase kinase-associated protein 1
81	SV2B	Synaptic vesicle glycoprotein 2B
82	TEFF2	Tomoregulin-2
83	THIO	Thioredoxin

Supplementary Data 2. Detected proteins in WT brain vessels		
**#**	**Accession**	**Description**
1	ACTA	Actin, aortic smooth muscle
2	PGBM	Basement membrane-specific heparan sulfate proteoglycan core protein
3	MYH11	Myosin-11
4	ACTN4	Alpha-actinin-4
5	TBB4B	Tubulin beta-4B chain
6	TPM1	Tropomyosin alpha-1 chain
7	ACTN1	Alpha-actinin-1
8	FLNA	Filamin-A
9	ACON	Aconitate hydratase, mitochondrial
10	K1C18	Keratin, type I cytoskeletal 18
11	ALDH2	Aldehyde dehydrogenase, mitochondrial
12	TPM2	Tropomyosin beta chain
13	TAGL	Transgelin
14	VINC	Vinculin
15	HS90B	Heat shock protein HSP 90-beta
16	THIM	3-ketoacyl-CoA thiolase, mitochondrial
17	CO4A2	Collagen alpha-2(IV) chain
18	LAMC1	Laminin subunit gamma-1
19	ENPL	Endoplasmin
20	LAMB2	Laminin subunit beta-2
21	SRBS2	Sorbin and SH3 domain-containing protein 2
22	LDHB	L-lactate dehydrogenase B chain
23	PDIA3	Protein disulfide-isomerase A3
24	ADT2	ADP/ATP translocase 2
25	LAMA5	Laminin subunit alpha-5
26	ECHB	Trifunctional enzyme subunit beta, mitochondrial
27	H3C	Histone H3.3C
28	H2B1B	Histone H2B type 1-B
29	TAGL2	Transgelin-2
30	ECHA	Trifunctional enzyme subunit alpha, mitochondrial
31	ETFB	Electron transfer flavoprotein subunit beta
32	S12A2	Solute carrier family 12 member 2
33	TTHY	Transthyretin
34	GPM6A	Neuronal membrane glycoprotein M6-a
35	COX2	Cytochrome c oxidase subunit 2
36	COX41	Cytochrome c oxidase subunit 4 isoform 1, mitochondrial
37	B3A2	Anion exchange protein 2
38	IDHP	Isocitrate dehydrogenase [NADP], mitochondrial
39	IVD	Isovaleryl-CoA dehydrogenase, mitochondrial
40	ML12B	Myosin regulatory light chain 12B
41	PCBP1	Poly(rC)-binding protein 1
42	SDHA	Succinate dehydrogenase [ubiquinone] flavoprotein subunit, mitochondrial
43	CO1A1	Collagen alpha-1(I) chain
44	ACADL	Long-chain specific acyl-CoA dehydrogenase, mitochondrial
45	AQP1	Aquaporin-1
46	ATP5H	ATP synthase subunit d, mitochondrial
47	ATPO	ATP synthase subunit O, mitochondrial
48	DDX5	Probable ATP-dependent RNA helicase DDX5
49	MYL9	Myosin regulatory light polypeptide 9
50	RL13	60S ribosomal protein L13
51	RL26	60S ribosomal protein L26
52	RL8	60S ribosomal protein L8
53	RS14	40S ribosomal protein S14
54	RS18	40S ribosomal protein S18
55	TGM2	Protein-glutamine gamma-glutamyltransferase 2
56	TINAL	Tubulointerstitial nephritis antigen-like
57	AT2B1	Plasma membrane calcium-transporting ATPase 1
58	QCR7	Cytochrome b-c1 complex subunit 7
59	ATP5J	ATP synthase-coupling factor 6, mitochondrial
60	PRDX3	Thioredoxin-dependent peroxide reductase, mitochondrial
61	RL7	60S ribosomal protein L7
62	ATP5I	ATP synthase subunit e, mitochondrial
63	BCAM	Basal cell adhesion molecule
64	CAVN1	Caveolae-associated protein 1
65	CO6A1	Collagen alpha-1(VI) chain
66	COIA1	Collagen alpha-1(XVIII) chain
67	EF1D	Elongation factor 1-delta
68	GTR1	Solute carrier family 2, facilitated glucose transporter member 1
69	NB5R3	NADH-cytochrome b5 reductase 3
70	NDUS1	NADH-ubiquinone oxidoreductase 75 kDa subunit, mitochondrial
71	NID1	Nidogen-1
72	PRELP	Prolargin
73	PTMA	Prothymosin alpha
74	RET1	Retinol-binding protein 1
75	RL3	60S ribosomal protein L3

Supplementary Data 3. Detecteed proteins in Aβ-positive vessels

#	Accession	Description
1	MFG-E8	Lactadherin
2	TIMP3	Metalloproteinase inhibitor 3

Supplementary Data 4. Common proteins between Aβ plaques and Aβ vessels

#	Accession	Description
1	APOE	Apolipoprotein E
2	A4	Amyloid-beta A4 protein
3	VTNC	Vitronectin
4	HTRA1	Serine protease HTRA1
5	TICN2	Testican-2
6	C1QB	Complement C1q subcomponent subunit B

Supplementary Data 5. Common proteins between WT brain vessels and Aβ vessels

#	Accession	Description
1	ACTA	Actin, aortic smooth muscle
2	PGBM	Basement membrane-specific heparan sulfate proteoglycan core protein
3	MYH11	Myosin-11
4	TPM1	Tropomyosin alpha-1 chain
5	FLNA	Filamin-A
6	TAGL	Transgelin
7	VINC	Vinculin
8	H12	Histone H1.2
9	H14	Histone H1.4
10	H2A2A	Histone H2A type 2-A
11	CO4A2	Collagen alpha-2(IV) chain
12	LAMC1	Laminin subunit gamma-1
13	LAMB2	Laminin subunit beta-2
14	SRBS2	Sorbin and SH3 domain-containing protein 2
15	LAMA5	Laminin subunit alpha-5
16	HBA	moglobin subunit alpha
17	CO1A1	Collagen alpha-1(I) chain
18	ROA3	Heterogeneous nuclear ribonucleoprotein A3
19	MYL9	Myosin regulatory light polypeptide 9
20	TINALE	Tubulointerstitial nephritis antigen-like
21	CO4A1	Collagen alpha-1(IV) chain
22	BCAM	Basal cell adhesion molecule
23	CO6A1	Collagen alpha-1(VI) chain
24	COIA1	Collagen alpha-1(XVIII) chain
25	NID1	Nidogen-1
26	HBB1	Hemoglobin subunit beta-1

Supplementary Data 6. Common proteins between Aβ plaques and WT brain vessels

#	Accession	Description
1	TBB4B	Tubulin beta-4B chain
2	ACTC	Actin, alpha cardiac muscle 1
3	TBA4A	Tubulin alpha-4A chain
4	AT1A1	Sodium/potassium-transporting ATPase subunit alpha-1
5	GNAO	Guanine nucleotide-binding protein G(o) subunit alpha
6	HS90B	Heat shock protein HSP 90-beta
7	SPTN1	Spectrin alpha chain, non-erythrocytic 1
8	CALM1	Calmodulin-1
9	PGK1	Phosphoglycerate kinase 1
10	ACON	Aconitate hydratase
11	HXK1	Hexokinase-1
12	MOES	Moesin
13	MDHC	Moesin
14	HS90A	Heat shock protein HSP 90-alpha
15	GPM6A	Neuronal membrane glycoprotein M6-a
16	PPIA	Peptidyl-prolyl cis-trans isomerase A
17	LDHB	L-lactate dehydrogenase B chain
18	TPIS_	Triosephosphate isomerase
19	AATM	Aspartate aminotransferase, mitochondrial
20	CLH1	Clathrin heavy chain 1
21	AT1B1	Sodium/potassium-transporting ATPase subunit beta-1
22	MDHM	Malate dehydrogenase, mitochondrial
23	GDIA	Rab GDP dissociation inhibitor alpha
24	LDHA	L-lactate dehydrogenase A chain
25	VATA	V-type proton ATPase catalytic subunit A
26	TERA	Transitional endoplasmic reticulum ATPase
27	DHE3	Glutamate dehydrogenase 1
28	ADT1	ADP/ATP translocase 1
29	ARF1	ADP-ribosylation factor 1
30	AT2B1	Plasma membrane calcium-transporting ATPase 1
31	SAP	Prosaposin
32	EFHD2	EF-hand domain-containing protein D2
33	GRP78	78 kDa glucose-regulated protein
34	GNAI2	Guanine nucleotide-binding protein G(i) subunit alpha-2
35	COF1	Cofilin-1
36	UBA1	Ubiquitin-like modifier-activating enzyme 1
37	ENPL	Endoplasmin
38	IDH3A	Isocitrate dehydrogenase [NAD] subunit alpha
39	MPCP	Phosphate carrier protein, mitochondrial
40	ANXA5	Annexin A5
41	THIL	Acetyl-CoA acetyltransferase
42	MIF	Macrophage migration inhibitory factor
43	NHRF1	Na(+)/H(+) exchange regulatory cofactor NHE-RF1
44	NDKA	V-type proton ATPase catalytic subunit A
45	ANXA3	Annexin A3
46	PDIA3	Protein disulfide-isomerase A3
47	CAH2	Carbonic anhydrase 2
48	TAGL2	Transgelin-2
49	COX2	Cytochrome c oxidase subunit 2
50	COX41	Cytochrome c oxidase subunit 4 isoform 1
51	ATPD	ATP synthase subunit delta
52	RTN4	Reticulon-4
53	EFTU	Elongation factor Tu
54	FKB1A	Peptidyl-prolyl cis-trans isomerase FKBP1A
55	PCP4	Calmodulin regulator protein PCP4
56	PROF1	Profilin-1
57	UBP5	Ubiquitin carboxyl-terminal hydrolase 5
58	MARCS	Myristoylated alanine-rich C-kinase substrate
59	TKT	Transketolase
60	DEST	Destrin
61	PLM	Phospholemman
62	SERA	D-3-phosphoglycerate dehydrogenase
63	CATB	Cathepsin B
64	COX5A	Cytochrome c oxidase subunit 5A
65	HINT1	Histidine triad nucleotide-binding protein 1
66	MTPN	Myotrophin
67	PSB6	Proteasome subunit beta type-6
68	SAHH2	S-adenosylhomocysteine hydrolase-like protein 1
69	GSTP1	Glutathione S-transferase P 1

Supplementary Data 7. Common proteins among the three experimental groups

#	Accession	Description
1	CLUS	Clusterin
2	TBB5	Tubulin beta-5 chain
3	TBB2A	Tubulin beta-2A chain
4	GFAP	Glial fibrillary acidic protein
5	AT1A2	Sodium/potassium-transporting ATPase subunit alpha-2
6	HSP7C	Heat shock cognate 71 kDa protein
7	KCRB	Creatine kinase B-type
8	VIME	Vimentin
9	DPYL2	Dihydropyrimidinase-related protein 2
10	ATPB	ATP synthase subunit beta, mitochondrial
11	ALDOA	Fructose-bisphosphate aldolase A
12	GSTM1	Glutathione S-transferase Mu 1
13	BASP1	Brain acid soluble protein 1
14	G3P	Glyceraldehyde-3-phosphate dehydrogenase
15	GLNA	Glutamine synthetase
16	ATPA	ATP synthase subunit alpha, mitochondrial
17	1433G	14-3-3 protein gamma
18	1433E	14-3-3 protein epsilon
19	ALDOC	Fructose-bisphosphate aldolase C
20	1433B	14-3-3 protein beta/alpha
21	ENOG	Gamma-enolase
22	PRDX6	Peroxiredoxin-6
23	1433T	14-3-3 protein theta
24	SPTB2	Spectrin beta chain, non-erythrocytic 1
25	MBP	Myelin basic protein
26	1433F	14-3-3 protein eta
27	PEBP1	Phosphatidylethanolamine-binding protein 1
28	AATC	Aspartate aminotransferase, cytoplasmic
29	H2A1B	Histone H2A type 1-B
30	SODC	Superoxide dismutase [Cu-Zn]
31	H3C	Histone H3.3C
32	H2B1B	Histone H2B type 1-B
33	MYH9	Myosin-9
34	VDAC1	Voltage-dependent anion-selective channel protein 1
35	ACTN1	Alpha-actinin-1
36	GDIR1	Rho GDP-dissociation inhibitor 1
