# Supplementary material for: MFG-E8 (LACTADHERIN): a novel marker associated with cerebral amyloid angiopathy
Source: Acta Neuropathol Commun. 2021 Sep 16;9:154. doi: 10.1186/s40478-021-01257-9 (PMC8444498; doi:10.1186/s40478-021-01257-9)
Supplement: Supplementary file 4 — Additional file 4. Cause of death and patient demographic and neuropathological characteristics in cases with and without CAA. [file 40478_2021_1257_MOESM4_ESM.pdf]

Cause of death and patient demographic and neuropathological characteristics in cases with and without CAA.

Case	Diagnosis	Age	Sex	CAA type[34]	Vonsattel score[33]	Braak tangle stage[36]	ABC[35]	AD probability (neuropathologic change)[35]	ICH	Cause of death
1	CAA	83	M	I	IV	III	A3B2C3	Yes (Intermediate)	No	-
2	CAA	81	F	I	III	III	A3B2C3	Yes (Intermediate)	Yes	ICH
3	CAA	73	M	I	II	I	A1B1C1	No (Low)	Yes	Infective endocarditis
4	CAA	93	F	II	I	II	A2B1C3	Yes (Low)	No	Mesenteric thrombosis
5	CAA	81	F	I	III	II	A2B1C1	Yes (Low)	No	Ischemic stroke
6	CAA	83	M	II	II	II	A3B1C2	Yes (Low)	No	-
7	Control	70	F	N/A	N/A	I	A0B1C0	No	No	PML
8	Control	75	M	N/A	N/A	I	A0B1C0	No	No	Metastatic lung cancer
9	Control	79	M	N/A	N/A	0	A1B0C2	No (Low)	Yes	ICH
10	Control	82	F	N/A	N/A	I	A2B1C1	No (Low)	No	Ischemic cardiomyopathy
11	Control	71	M	N/A	N/A	I	A0B1C0	No	No	Multiple myeloma
12	Control	70	F	N/A	N/A	0	A1B0C0	No (Low)	No	Bacterial pneumonia

Abbreviations: F, female; M, male; CAA, Cerebral Amyloid Angiopathy; AD, Alzheimer's disease; ICH, Intracerebral hemorrhage; N/A, not applicable; -, not known; PML, Progressive multifocal leukoencephalopathy.
