# Supplementary material for: MFG-E8 (LACTADHERIN): a novel marker associated with cerebral amyloid angiopathy
Source: Acta Neuropathol Commun. 2021 Sep 16;9:154. doi: 10.1186/s40478-021-01257-9 (PMC8444498; doi:10.1186/s40478-021-01257-9)
Supplement: Supplementary file 5 — Additional file 5. Univariate analysis of MFG-E8 distribution according to the demographic and neuropathological characteristics of the CAA brain cohort. [file 40478_2021_1257_MOESM5_ESM.pdf]

Univariate analysis of MFG-E8 distribution according to the demographic and neuropathological characteristics of the CAA brain cohort.

Variable	*p*-Value
Age	0.151
Sex	0.369
ABC	0.256
Braak	0.197
CAA type	0.075
Vonsattel	0.265
ICH	0.264

Abbreviations: ICH, Intracerebral hemorrhage.
