# Supplementary material for: MFG-E8 (LACTADHERIN): a novel marker associated with cerebral amyloid angiopathy
Source: Acta Neuropathol Commun. 2021 Sep 16;9:154. doi: 10.1186/s40478-021-01257-9 (PMC8444498; doi:10.1186/s40478-021-01257-9)
Supplement: Supplementary file 6 — Additional file 6. Demographic and clinical characteristics of healthy controls, CAA-ICH patients, and AD patients. [file 40478_2021_1257_MOESM6_ESM.pdf]

Demographic and clinical characteristics of healthy controls, CAA-ICH patients, and AD patients.

Variable	Control (n=39)	CAA-ICH (n=31)	AD (n=25)	*p*-Value
Age, years, median (IQR)	75 (71-78.5)	77 (71.5-80)	80 (75-82)	0.069
Sex (female), n (%)	20 (51.3%)	16 (51.6%)	19 (76%)	0.102
Hypertension, n (%)	18 (60%)	15 (53.6%)	16 (64%)	0.736
Diabetes, n (%)	5 (16.7%)	6 (24%)	5 (20%)	0.795
Dyslipidemia, n (%)	10 (33.3%)	5 (22.7%)	-	0.404
Serum MFG-E8, pg/ml, median (IQR)	2636.6 (1959.6-3912.1)	2099.3 (1755.1-3053.9)	2488.9 (1878.9-3318.5)	0.184

Abbreviations: CAA-ICH, Cerebral amyloid angiopathy-associated intracerebral hemorrhage; AD, Alzheimer's disease; IQR, interquartile range; -, not known.
