# Supplementary material for: MFG-E8 (LACTADHERIN): a novel marker associated with cerebral amyloid angiopathy
Source: Acta Neuropathol Commun. 2021 Sep 16;9:154. doi: 10.1186/s40478-021-01257-9 (PMC8444498; doi:10.1186/s40478-021-01257-9)
Supplement: Supplementary file 7 — Additional file 7. Univariate analysis of circulating MFG-E8 levels according to demographic and clinical characteristics of the total cohort. [file 40478_2021_1257_MOESM7_ESM.pdf]

Univariate analysis of circulating MFG-E8 levels according to demographic and clinical characteristics of the total cohort.

**Total Cohort (n=95)**	**Serum MFG-E8 levels (pg/ml)**		
**Variable**	**Yes**	**No**	***p*-Value**
Age	r= - 0.061		0.556
Sex (female)	2382.5 (1879.1-3198.4)<br>n=55	2594.6 (1719.6-3973.6)<br>n=40	0.667
Hypertension	2488.9 (1878.9-3237.1)<br>n=49	2120.1 (1666.2-3194.9)<br>n=34	0.517
Diabetes	2794.7 (2322.6-3302.1)<br>n=16	2151.6 (1695.7-3198.4)<br>n=64	0.095
Dyslipidemia	2313.6 (1845.2-3518.4)<br>n=15	2162.4 (1774.7-3042.3)<br>n=37	0.525

Data are expressed as median picograms per milliliter (interquartile range). r, Spearman's rho correlation.
