# Supplementary material for: MFG-E8 (LACTADHERIN): a novel marker associated with cerebral amyloid angiopathy
Source: Acta Neuropathol Commun. 2021 Sep 16;9:154. doi: 10.1186/s40478-021-01257-9 (PMC8444498; doi:10.1186/s40478-021-01257-9)
Supplement: Supplementary file 8 — Additional file 8. Western blot and qPCR results showing the gene silencing efficiency of the siRNA sequence targeting MFG-E8 in HBVSMCs. [file 40478_2021_1257_MOESM8_ESM.pdf]

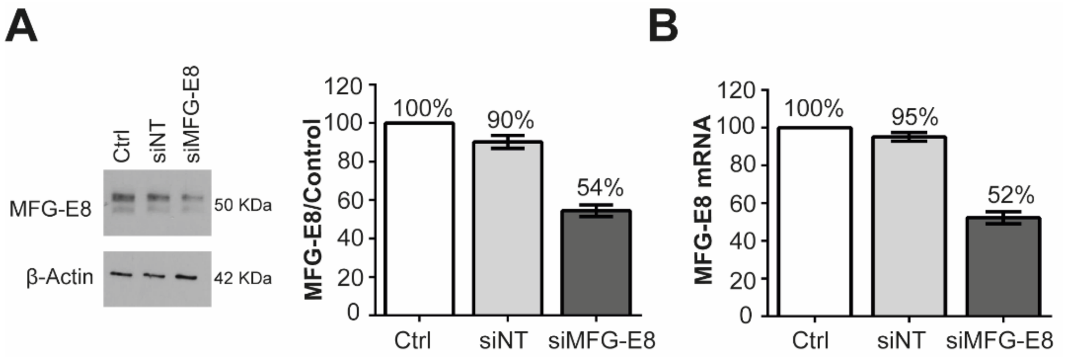

Western blot (A) and qPCR (B) results showing the gene silencing efficiency of the siRNA sequence targeting MFG-E8 in HBVSMCs. HBVSMCs were treated with 1 µM MFG-E8 siRNA or non-targeting siRNA for 48 hours. Ctrl, control; siNT, non-targeting siRNA.
